# A Markovian SuASR Epidemic Framework With Stochastic External Factors

**DOI:** 10.1002/sim.70796

**Published:** 2026-11-09

**Authors:** Vasileios E. Papageorgiou, Georgios Vasiliadis

**Affiliations:** ^1^ Department of Mathematics Aristotle University of Thessaloniki Thessaloniki Greece; ^2^ Department of Mathematics University of Western Macedonia Kastoria Greece

**Keywords:** joint distributions, Markov processes, path‐dependent functionals, probability generating functions, random factors

## Abstract

Epidemic waves are often shaped by uncertain external drivers, such as environmental variability or behavioral and policy changes, that can randomly amplify or dampen transmission. Capturing this randomness requires models whose dynamics evolve under time‐varying, stochastic influences. In this paper, we introduce a stochastic susceptible–asymptomatic–symptomatic–removed (SuASR) framework that embeds random external factors directly into the epidemic mechanism through an extended continuous‐time Markov chain (CTMC). The proposed framework is flexible enough to represent epidemic dynamics both with reinfection and with infection‐induced immunity, depending on the epidemiological setting under study. Unlike standard Markovian epidemic models, our formulation explicitly tracks two concurrent infectious sources (asymptomatic and symptomatic), yielding coupled infection pathways and substantially richer transient behavior. A key contribution is analytical tractability, where we derive computable expressions for the joint distribution of asymptomatic and symptomatic infection‐event counts and develop tools tailored to extreme‐outbreak risk, including the distribution of the maximum infectious burden. These results enable probabilistic quantification of epidemic severity beyond mean trajectories, providing direct estimates of the likelihood of rare but consequential surges. We validate the model through an extensive sensitivity analysis and an application to the H1N1 influenza epidemic, illustrating how stochastic external influences reshape outbreak profiles, total infections from both sources, and the probability of extreme epidemic scenarios.

## 1 Introduction

The modeling of infectious diseases based on advanced mathematical models has flourished during the last decades where more advanced deterministic and stochastic approaches are proposed. Deterministic methods, utilizing non‐linear systems of differential equations, like the SIR model, aim to predict epidemic trajectories efficiently [1, 2, 3]. Each letter corresponds to one of the model's compartments as the population is assumed to be partitioned into three classes/states, namely the susceptible (S), infectious (I), and recovered (R). Several extensions have been proposed which account for the existence of exposed individuals [4, 5], deaths [6, 7], vaccinations [8], or additional control measures [9, 10, 11]. However, the aforementioned deterministic approaches struggle to capture the time‐varying dynamics of epidemics, while the stability analysis of epidemic equilibria based on compartmental schemes with constant parameters is not always informative.

In contrast, stochastic modeling is useful for capturing the effects of demographic, transmission, and environmental variability [12, 13, 14]. These methods often predict epidemic spread using Bayesian estimation techniques, such as Kalman and particle filters [15, 16, 17, 18, 19, 20, 21, 22, 23]. Other studies focus on stochastic descriptors, offering insights about the progression of the phenomenon. Examples include time‐ and size‐related features such as the maximum number of infectious cases [24], the time of infection or mortality [12, 25], the disease extinction duration [26], the quasi‐stationarity of the system [24, 27, 28], and so forth. Some research delves into vaccine‐focused approaches, determining optimal revaccination times or quantifying total infectious cases [29, 30, 31].

Moreover, related stochastic epidemic models have already addressed several final‐outcome quantities. Ball and Clancy studied final size and severity in stochastic multitype epidemic models [32], while Ball and O'Neill derived exact results for final size and more general final‐state random variables [33]. Other works have considered symptomatic and asymptomatic infectives, Markovian random environments, and computational methods for final‐outcome or final‐size distributions [27, 34, 35]. More recently, symptomatic and asymptomatic infectious classes have also been studied under semi‐Markov switching [36].

It is known that there are many epidemics which are characterized by varying parameters and more specifically seasonal changes, like measles, diphtheria, chickenpox, cholera, or influenza‐type viruses [37]. These changes may arise from environmental factors, such as temperature or humidity, or from the implementation of public health measures. Moreover, the variability of parameters, particularly the timing of transitions between environmental states, is marked by uncertainty, as the emergence of a new variant or the impact of a vaccination campaign or lockdown cannot be predicted with absolute precision. Given this, deterministic methods may be inadequate. Therefore, the development of new stochastic approaches that can capture both parameter variability and uncertainty is essential.

In this paper, we introduce a stochastic *Su*sceptible–*A*symptomatic–*S*ymptomatic–*R*emoved (SuASR) epidemic model which considers the existence of random external factors, based on an extended CTMC. Random factors may be influenced by social patterns, climatic variables, or the establishment of control measures like vaccination or lockdowns which can significantly alter the parameters that dictate the prevalence of the infectious disease [38, 39]. Existing literature mainly focuses on models where environmental changes are either not taken into account or are assumed to be deterministic. For instance, the prevalent assumption involves disease transmission following a sinusoidal function, indicating that the contact rate fluctuates over time but without considering uncertainty [40, 41, 42, 43]. However, there is limited attention given to models incorporating stochastic variations in the environment. Indicatively, we refer to the papers [27, 44] and [45], where the authors explore epidemic models with random environment, employ a cyclically alternating renewal process according to the specificities of the SIR scheme and introduce a deterministic SIS model with Markovian environment, correspondingly.

Compared to the aforementioned studies, our model accounts for two sources of infection (arising from both asymptomatic and symptomatic cases) thereby capturing more complex and realistic epidemic dynamics. The proposed framework can accommodate epidemic dynamics both with reinfection (Variant I) and with infection‐induced immunity (Variant II), according to the epidemiological context under consideration.

Additionally, we derive formulas for stochastic descriptors that are relevant to epidemic severity, focusing on both cumulative infection burden and extreme transmission events. In particular, we study the joint distribution of symptomatic and asymptomatic infection‐event counts, as well as the distribution of the peak number of infectious individuals, using a suitably modified Markov chain. Thus, our contribution is the matrix‐recursive computation of these distributional quantities for a finite‐population SuASR model whose parameters vary according to external factor states (EFSs).

Efficient computational schemes are introduced to compute joint and marginal distributions, along with their moments. A comprehensive sensitivity analysis is conducted to explore the model's behavior in relation to key parameters. To further demonstrate the credibility and practical value of the proposed stochastic framework, we present a parameter‐based scenario analysis motivated by the 2009 H1N1 influenza pandemic in Mexico. Finally, we offer insights into efficient computational strategies for evaluating the proposed stochastic descriptors.

Overall, the main novel contributions of this analysis are: (a) A new Markovian epidemic model (SuASR) incorporating both symptomatic and asymptomatic cases, (b) a stochastic process with time‐varying parameters accounting for random external factors, (c) formulas for efficiently computing the joint distribution of symptomatic and asymptomatic cases, (d) expressions for estimating the maximum number of simultaneously infectious cases, (e) demonstration of the model's behavior through sensitivity analysis and a parameter‐based H1N1 scenario analysis.

## 2 Methodology

We introduce a novel stochastic epidemiological model that accounts for the existence of random external factors. These factors may relate to environmental elements such as temperature or humidity, emergence of new disease strains, public health interventions, and more. Incorporating these random factors stands as a primary innovation in our analysis, allowing for the accommodation of inherent variations within natural phenomena. Initially, we outline a deterministic compartmental framework before transitioning to its stochastic counterpart. After that, a series of important epidemiological descriptors is provided, examining the joint distribution of symptomatic and asymptomatic cases and the maximum infectious burden.

### 2.1 The Stochastic SuASR Framework With Random External Factors

We introduce a stochastic SuASR framework with random external factors through an extended CTMC. To accommodate different epidemiological settings, we consider two related formulations that share the same stochastic architecture but differ in the way recovery is modeled. The two variants share the same state space architecture and differ only in the way individuals are routed after infection. We first present the recurrent‐infection formulation and then describe the immunity‐preserving formulation used when reinfections are not expected within the outbreak horizon.

#### 2.1.1 Variant I: Recurrent‐Infection Formulation

Before delving into the stochastic epidemic model featuring random external factors, it is important to outline the foundational epidemiological framework upon which we build the stochastic equivalent model. The deterministic SuASR framework consists of a system of four ordinary differential equations (ODEs), describing the evolution in the number of susceptible, asymptomatic, symptomatic, and removed cases. The corresponding compartmental structure is illustrated in Equation (1), where

(1) $$\begin{array}{cc} \frac{dS(t)}{dt} & =-\frac{\beta }{N}S(t)\left(I(t)+A(t)\right)+\gamma I(t)+\kappa A(t),\\ \frac{dA(t)}{dt} & =(1-p)\frac{\beta }{N}S(t)\left(I(t)+A(t)\right)-\kappa A(t),\\ \frac{dI(t)}{dt} & =p\frac{\beta }{N}S(t)\left(I(t)+A(t)\right)-\gamma I(t)-\mu I(t),\\ \frac{dR(t)}{dt} & =\mu I(t), \end{array}$$

for t≥0. The term N denotes the population size, β the disease's transmission/infection rate, p the ratio of symptomatic infections, μ the removal rate and γ, and κ the recovery rates from a symptomatic or an asymptomatic infection, respectively. Moreover, S(t), I(t), A(t), and R(t) represent the number of susceptible, symptomatic, asymptomatic, and removed cases at time t.

Recovery from asymptomatic or symptomatic infection returns individuals to the susceptible class, so infection does not necessarily confer immunity in this formulation. Since the rate μ may be associated with disease‐induced removal of symptomatic individuals, we include the corresponding transition only from the symptomatic compartment to the removed class. This choice is justified by the (almost) negligible mortality rate among asymptomatic cases, as the absence of symptoms typically indicates less severe infections. Moreover, the population under study is assumed to be closed, with homogeneous contact patterns among individuals.

Now, for the transition to the stochastic point of view, we employ a CTMC, namely 𝒳={X(t)=(I(t),A(t),R(t)),t≥0}. The fourth state of system (1) can be described as a linear combination of the three remaining states, that is, S(t)=N−I(t)−A(t)−R(t), for t≥0. Considering that the Markov chain is in the initial/current state (i,a,r) at the time t, the transition rates of the Markov chain are given by expression (2), where

(2) $$u_{\left(i,a,r),(i^{\prime },a^{\prime },\;r^{\prime }\right)}=\left\{\begin{array}{ll} -u_{i,a,r},\; & (i^{\prime },a^{\prime },r^{\prime })=(i,a,r),\\ p{\left(\frac{\beta }{N}\right)}_{i,a,r},\; & (i^{\prime },a^{\prime },r^{\prime })=(i+1,a,r),\\ (1-p){\left(\frac{\beta }{N}\right)}_{i,a,r},\; & (i^{\prime },a^{\prime },r^{\prime })=(i,a+1,r),\\ \gamma_{i},\; & (i^{\prime },a^{\prime },r^{\prime })=(i-1,a,r),\\ \kappa_{a},\; & (i^{\prime },a^{\prime },r^{\prime })=(i,a-1,r),\\ \mu_{i},\; & (i^{\prime },a^{\prime },r^{\prime })=(i-1,a,r+1),\\ 0,\; & \text{otherwise}, \end{array}\right.$$

while $p{\left(\frac{\beta }{N}\right)}_{i,a,r}=p\left(\frac{\beta }{N}\right)(i+a)(N-i-a-r)$, $\left(1-p){\left(\frac{\beta }{N}\right)}_{i,a,r}=(1-p)(\frac{\beta }{N})(i+a)(N-i-a-r\right)$, $\gamma_{i}=\gamma i$, $\kappa_{a}=\kappa a$, $\mu_{i}=\mu i$ and $u_{i,a,r}=p\left(\frac{\beta }{N}\right)(i+a)(N-i-a-r)+(1-p)\left(\frac{\beta }{N}\right)(i+a)(N-i-a-r)+\gamma i+\kappa a+\mu i$.

The form of Equation (2) derives from the fact that we have five possible transitions assuming that we initiate the examination of the phenomenon from the transient state (i,a,r). Ultimately, the Markov chain 𝒳 transitions from state (i,a,r), to the state

i. (i,a+1,r), with rate ($1-p){\left(\frac{\beta }{N}\right)}_{i,a,r}$, when a susceptible case becomes asymptomatic,
ii. (i+1,a,r), with rate $p{\left(\frac{\beta }{N}\right)}_{i,a,r}$, when a susceptible case becomes symptomatic,
iii. (i−1,a,r), with rate $\gamma_{i}$, when a symptomatic case recovers,
iv. (i,a−1,r), with rate $\kappa_{a}$, when an asymptomatic case recovers,
v. (i−1,a,r+1), with rate $\mu_{i}$, when a symptomatic individual is removed.

The infinitesimal generator matrix $U=(u_{\left(i,a,r),(i^{\prime },a^{\prime },r^{\prime }\right)}:\;(i,a,r),(i^{\prime },a^{\prime },r^{\prime })\in A_{U})$, is a square matrix having a block tridiagonal form. The corresponding state space $A_{U}=\{(i,a,r):0\leq i\leq N,\;0\leq a\leq N-i,\;0\leq r\leq N-i-a\}$ contains a total of $\left|A_{U}\right|=\sum_{i=0}^{N}\sum_{a=0}^{N-i}(N-i-a+1)=\frac{\left(N+1)(N+2)(N+3\right)}{6}$ states.

As we mentioned in the Introduction, one of the main objectives of the present analysis is the development of an epidemic model that accounts for external random factors, which can alter the parameters that determine the progression of the disease. This mechanism is governed by the CTMC ℱ={F(t),t≥0} with state space $A_{F}=\{f:1\leq f\leq F\}$ and transition rates $u_{F}(f,f^{\prime })$, for $1\leq f,f^{\prime }\leq F$. This construction represents external changes, such as environmental variation or externally imposed policy regimes, rather than interventions triggered by the current number of infectious individuals. If the total exit rate from an EFS is positive, then the holding time is exponentially distributed with mean equal to the reciprocal of the sum of all transition rates leaving that state. If the total exit rate is zero, then the EFS is absorbing for the external‐factor process.

We propose an extension of the Markov chain 𝒳 by adding a fourth component, which is associated with a finite number of EFSs. We introduce the Markov chain $\tilde{𝒳}=\left\{\tilde{X}\left(t\right)=\left(I\left(t\right),A\left(t\right),R\left(t\right),F\left(t\right)\right),t\geq 0\right\}$. An extension of the state space is also dictated, where $A=A_{U}\times A_{F}=\left\{\left(i,a,r,f\right):0\leq i\leq N,\;0\leq a\leq N-i,\;0\leq r\leq N-i-a,\;1\leq f\leq F\right\}$. Thus, the cardinality of the extended state space is $\left|A\right|=\left|A_{F}\right|\cdot \left|A_{U}\right|=F\frac{\left(N+1\right)\left(N+2\right)\left(N+3\right)}{6}$. In parallel, a new infinitesimal generator matrix $\tilde{U}=\left\{{\tilde{u}}_{\left(i,a,r,f\right),\left(i^{\prime },a^{\prime },r^{\prime },f^{\prime }\right)}:\left(i,a,r,f\right),\left(i^{\prime },a^{\prime },r^{\prime },f^{\prime }\right)\in A\right\}$ is constructed, where

(3) $$\begin{array}{cc} & {\tilde{u}}_{\left(i,a,r,f\right),\left(i^{\prime },a^{\prime },r^{\prime },f^{\prime }\right)}\\ & \;=\left\{\begin{array}{ll} -{\tilde{u}}_{i,a,r,f},\; & \left(i^{\prime },a^{\prime },r^{\prime },f^{\prime }\right)=\left(i,a,r,f\right),\\ p\left(f\right){\left(\frac{\beta \left(f\right)}{N}\right)}_{i,a,r},\; & \left(i^{\prime },a^{\prime },r^{\prime },f^{\prime }\right)=\left(i+1,a,r,f\right),\\ \left(1-p\left(f\right)\right){\left(\frac{\beta \left(f\right)}{N}\right)}_{i,a,r},\; & \left(i^{\prime },a^{\prime },r^{\prime },f^{\prime }\right)=\left(i,a+1,r,f\right),\\ \gamma_{i}\left(f\right),\; & \left(i^{\prime },a^{\prime },r^{\prime },f^{\prime }\right)=\left(i-1,a,r,f\right),\\ \kappa_{a}\left(f\right),\; & \left(i^{\prime },a^{\prime },r^{\prime },f^{\prime }\right)=\left(i,a-1,r,f\right),\\ \mu_{i}\left(f\right),\; & \left(i^{\prime },a^{\prime },r^{\prime },f^{\prime }\right)=\left(i-1,a,r+1,f\right),\\ u_{F}\left(f,f^{\prime }\right),\; & \left(i^{\prime },a^{\prime },r^{\prime },f^{\prime }\right)=\left(i,a,r,f^{\prime }\right),f\neq f^{\prime },\\ 0,\; & \text{otherwise}. \end{array}\right. \end{array}$$

while ${\tilde{u}}_{i,a,r,f}=p\left(f\right){\left(\frac{\beta \left(f\right)}{N}\right)}_{i,a,r}+\left(1-\;p\left(f\right)\right){\left(\frac{\beta \left(f\right)}{N}\right)}_{i,a,r}+\gamma_{i}\left(f\right)+\kappa_{a}\left(f\right){+\mu }_{i}\left(f\right)+\sum_{f^{\prime }=1}^{F}u_{F}\left(f,f^{\prime }\right)$, for $f\neq f^{\prime }\in A_{F}.$ Term $u_{F}\left(f,f^{\prime }\right)$ denotes the transition rate from EFS f to $f^{\prime }\neq f\in A_{F}$.

The state space A is divided into three nested levels, namely, $A={⋃}_{i=0}^{N}l(i)$, where the l(i) represents the first sublevel which is associated with I(t)=i. Furthermore, $l(i)={⋃}_{a=0}^{N-i}l(i,a)$ and $l(i,a)={⋃}_{r=0}^{N-i-a}l(i,a,r)$, where l(i,a,r)={(i,a,r,f):1≤f≤F}. The sets l(i,a) and l(i,a,r) represent the second and third level, correspondingly. The respective cardinalities are |l(i,a,r)|=F, |l(i,a)|=F(N−i−a+1) and $\left|l(i)\right|=\sum_{a=0}^{N-i}F(N-i-a+1)=F\frac{\left(N-i+1)(N-i+2\right)}{2}$. Finally, according to the aforementioned structure, l(0,0) represents a closed set of disease‐free states as there are no symptomatic or asymptomatic individuals who can spread the virus or trigger a secondary infection wave. Therefore, when the process enters the set l(0,0) the examination of the phenomenon terminates. This formulation is intended for epidemic settings in which repeated infections may occur over the time horizon of interest, and it is the version used in the sensitivity analyses of Sections 3.1 and 3.2.

#### 2.1.2 Variant II: Immunity‐Preserving Formulation

As noted in the Introduction, the removed state may include individuals who are either deceased or have acquired immunity following infection. For epidemic settings in which infected individuals develop complete immunity, or in which immunity may be imperfect but the observation window is too short for reinfections to occur, we introduce a modified version of the Markov chain defined in Equation (2). In this case, the infinitesimal generator matrix takes the following form

$$u_{\left(i,a,r),(i^{\prime },a^{\prime },r^{\prime }\right)}=\left\{\begin{array}{ll} -u_{i,a,r},\; & (i^{\prime },a^{\prime },r^{\prime })=(i,a,r),\\ p{\left(\frac{\beta }{N}\right)}_{i,a,r},\; & (i^{\prime },a^{\prime },r^{\prime })=(i+1,a,r),\\ (1-p){\left(\frac{\beta }{N}\right)}_{i,a,r},\; & (i^{\prime },a^{\prime },r^{\prime })=(i,a+1,r),\\ \kappa_{a},\; & (i^{\prime },a^{\prime },r^{\prime })=(i,a-1,r+1),\\ {\left(\gamma +\mu \right)}_{i},\; & (i^{\prime },a^{\prime },r^{\prime })=(i-1,a,r+1),\\ 0,\; & \text{otherwise}. \end{array}\right.$$

The key modification, relative to Equation (2), lies in the recovery transitions. Infected individuals now move only to the removed class rather than returning to the susceptible class. The extension of the process to incorporate random external factors through the addition of a fourth component can be carried out exactly as in Section 2.1.1. Under this formulation, fewer infections are expected, since recovered individuals enter the removed state and no longer participate in disease transmission.

For the remainder of the paper, the recursive forms developed in Sections 2.2 and 2.3 remain valid for Variant II after replacing the generator and all associated submatrices by their immunity‐preserving counterparts. In particular, the recovery transitions in the relevant generator blocks now lead to the removed class rather than to the susceptible class. In the compact matrix formulation, this affects the matrices in Equation (20), while for the maximum infectious burden it affects the transition structure in Equation (23). This immunity‐preserving formulation is employed in Section 3.3 for the H1N1 application.

### 2.2 The Number of Symptomatic and Asymptomatic Infections

At this point, we aim to construct formulas for the computation of the joint distribution of the number of symptomatic $\left(N^{S}\right)$ and asymptomatic $\left(N^{A}\right)$ infections until the eradication of the disease (total number). Thus, we examine the aforementioned stochastic descriptor until the Markov chain enters the set l(0,0). Let, T=inf{t≥0:(I(t),A(t),R(t),F(t))=(0,0,r,f),0≤r≤N,1≤f≤F} be the random variable which is associated with the extinction of the disease due to the absence of active infectious cases.

For both variants, the cumulative number of infection events until extinction is finite under the parameter conditions considered in this paper. Indeed, the CTMC has a finite epidemic state space and the disease‐free set is absorbing. Moreover, when the recovery and removal rates are positive, no closed communicating class exists outside the disease‐free set. Hence, absorption into the disease‐free set occurs with probability one, and the extinction time is finite almost surely. In Variant I, the same individual may be infected more than once, so the cumulative infection‐event counts are not necessarily bounded by N, but they remain finite almost surely.

The following theorem describes the computation of the joint distribution of the number of symptomatic/asymptomatic infections, denoted by ${\tilde{p}}_{i,a,r,f}(n,m)$, for $(n,m)\in {\left(\mathbb{N}\cup \left\{0\right\}\right)}^{2}$ and (i,a,r,f)∈A. Specifically, ${\tilde{p}}_{i,a,r,f}(n,m)$ denotes the probability that n new symptomatic and m asymptomatic cases will occur until disease's extinction. For the purposes of the next theorem, we assemble the corresponding probabilities into the column vector ${\tilde{p}}_{i,a}(n,m)$, where i, a, n, and m are considered fixed. The subscripts i and a refer to the initial numbers of symptomatic and asymptomatic infections, respectively. It should be noted that n and m refer to the number of infections occurring from the time at which the analysis begins until the extinction of the disease, excluding the initial number of symptomatic and asymptomatic cases.

Theorem 1 *The joint distribution of the number of symptomatic/asymptomatic cases until the disease's extinction can be computed through Equations* ((4), (5), (6), (7), (8), (9), (10), (11), (12), (13), (14), (15), (16), (17)). *For* n=m=0, *we have*

(4) $${\tilde{p}}_{0,0}(0,0)=1_{\left|l(0,0)\right|},$$

*while*

(5) $${\tilde{p}}_{0,0}(n,m)=0_{\left|l(0,0)\right|},\;(n,\;m)\neq (0,0),$$

*when there are no infected* (*symptomatic or asymptomatic*) *cases in the initial state. Now, when* i≠0 *or* a≠0, *we obtain*

(6) $${\tilde{p}}_{i,0}(0,0)=-{\tilde{U}}_{\left(i,0),(i,0\right)}^{-1}{\tilde{U}}_{\left(i,0),(i-1,0\right)}{\tilde{p}}_{i-1,0}(0,0),\;1\leq i\leq N,$$

(7) $${\tilde{p}}_{0,a}(0,0)=-{\tilde{U}}_{\left(0,a),(0,a\right)}^{-1}{\tilde{U}}_{\left(0,a),(0,a-1\right)}{\tilde{p}}_{0,a-1}(0,0),\;1\leq a\leq N,$$

*while when both indices are non‐zero, we obtain*

(8) $$\begin{array}{cc} {\tilde{p}}_{i,a}(0,0) & =-{\tilde{U}}_{\left(i,a),(i,a\right)}^{-1}\left[{\tilde{U}}_{\left(i,a),(i-1,a\right)}{\tilde{p}}_{i-1,a}(0,0)\right.\left.+\;{\tilde{U}}_{\left(i,a),(i,a-1\right)}{\tilde{p}}_{i,a-1}(0,0)\right],\\ & \;1\leq i\leq N-1,\;1\leq a\leq N-i. \end{array}$$

*Also, when* n>0,

(9) $$\begin{array}{cc} {\tilde{p}}_{i,0}(n,0) & =-{\tilde{U}}_{\left(i,0),(i,0\right)}^{-1}\left[\left(1-\delta_{N-i,0}\right){\tilde{U}}_{\left(i,0),(i+1,0\right)}{\tilde{p}}_{i+1,0}(n-1,0)\right.\\ & \;+\left.\;{\tilde{U}}_{\left(i,0),(i-1,0\right)}{\tilde{p}}_{i-1,0}(n,0)\right],\;1\leq i\leq N, \end{array}$$

(10) $$\begin{array}{cc} {\tilde{p}}_{0,a}(n,0) & =-{\tilde{U}}_{\left(0,a),(0,a\right)}^{-1}\left[\left(1-\delta_{N-a,0}\right){\tilde{U}}_{\left(0,a),(1,a\right)}{\tilde{p}}_{1,a}(n-1,0)\right.\\ & \;+\left.\;{\tilde{U}}_{\left(0,a),(0,a-1\right)}{\tilde{p}}_{0,a-1}(n,0)\right],\;1\leq a\leq N, \end{array}$$

*and*

(11) $$\begin{array}{cc} {\tilde{p}}_{i,a}(n,0) & =-{\tilde{U}}_{\left(i,a),(i,a\right)}^{-1}\left[\left(1-\delta_{N-a-i,0}\right){\tilde{U}}_{\left(i,a),(i+1,a\right)}{\tilde{p}}_{i+1,a}(n-1,0)\right.\\ & \;+{\tilde{U}}_{\left(i,a),(i-1,a\right)}{\tilde{p}}_{i-1,a}(n,0)\left.+{\tilde{U}}_{\left(i,a),(i,a-1\right)}{\tilde{p}}_{i,a-1}(n,0)\right],\\ & \;1\leq i\leq N-1,\;1\leq a\leq N-i. \end{array}$$

*On the other hand, when* m>0 *and* n=0, *we obtain*

(12) $$\begin{array}{cc} {\tilde{p}}_{i,0}(0,m) & =-{\tilde{U}}_{\left(i,0),(i,0\right)}^{-1}\left[\left(1-\delta_{N-i,0}\right){\tilde{U}}_{\left(i,0),(i,1\right)}{\tilde{p}}_{i,1}(0,m-1)\right.\\ & \;\left.+\;{\tilde{U}}_{\left(i,0),(i-1,0\right)}{\tilde{p}}_{i-1,0}(0,m)\right],\;1\leq i\leq N, \end{array}$$

(13) $$\begin{array}{cc} {\tilde{p}}_{0,a}(0,m) & =-{\tilde{U}}_{\left(0,a),(0,a\right)}^{-1}\left[\left(1-\delta_{N-a,0}\right){\tilde{U}}_{\left(0,a),(0,a+1\right)}{\tilde{p}}_{0,a+1}(0,m-1)\right.\\ & \;+\left.{\tilde{U}}_{\left(0,a),(0,a-1\right)}{\tilde{p}}_{0,a-1}(0,m)\right],\;1\leq a\leq N, \end{array}$$

*and*

(14) $$\begin{array}{cc} {\tilde{p}}_{i,a}(0,m) & =-{\tilde{U}}_{\left(i,a),(i,a\right)}^{-1}\left[\left(1-\delta_{N-a-i,0}\right){\tilde{U}}_{\left(i,a),(i,a+1\right)}{\tilde{p}}_{i,a+1}(0,m-1)\right.\\ & \;+{\tilde{U}}_{\left(i,a),(i-1,a\right)}{\tilde{p}}_{i-1,a}(0,m)+\left.{\tilde{U}}_{\left(i,a),(i,a-1\right)}{\tilde{p}}_{i,a-1}(0,m)\right],\\ & \;1\leq i\leq N-1,\;1\leq a\leq N-i. \end{array}$$

*Finally, when* m>0 *and* n>0, *we obtain*

(15) $$\begin{array}{cc} {\tilde{p}}_{i,0}(n,m) & =-{\tilde{U}}_{\left(i,0),(i,0\right)}^{-1}\left[\left(1-\delta_{N-i,0}\right){\tilde{U}}_{\left(i,0),(i+1,0\right)}{\tilde{p}}_{i+1,0}(n-1,m)\right.\\ & \;+\left(1-\delta_{N-i,0}\right){\tilde{U}}_{\left(i,0),(i,1\right)}{\tilde{p}}_{i,1}(n,m-1)\\ & \;\left.+\;{\tilde{U}}_{\left(i,0),(i-1,0\right)}{\tilde{p}}_{i-1,0}(n,m)\right],\;1\leq i\leq N, \end{array}$$

(16) $$\begin{array}{cc} {\tilde{p}}_{0,a}(n,m) & =-{\tilde{U}}_{\left(0,a),(0,a\right)}^{-1}\left[\left(1-\delta_{N-a,0}\right){\tilde{U}}_{\left(0,a),(1,a\right)}{\tilde{p}}_{1,a}(n-1,m)\right.\\ & \;+\left(1-\delta_{N-a,0}\right){\tilde{U}}_{\left(0,a),(0,a+1\right)}{\tilde{p}}_{0,a+1}(n,m-1)\\ & \;\left.+\;{\tilde{U}}_{\left(0,a),(0,a-1\right)}{\tilde{p}}_{0,a-1}(n,m)\right],\;1\leq a\leq N, \end{array}$$

*and*

(17) $$\begin{array}{cc} {\tilde{p}}_{i,a}\left(n,m\right) & \;=-{\tilde{U}}_{\left(i,a\right),\left(i,a\right)}^{-1}[\left(1-\delta_{N-a-i,0}\right){\tilde{U}}_{\left(i,a\right),\left(i+1,a\right)}{\tilde{p}}_{i+1,a}\left(n-1,m\right)\\ & \;+\left(1-\delta_{N-a-i,0}\right){\tilde{U}}_{\left(i,a\right),\left(i,a+1\right)}{\tilde{p}}_{i,a+1}\left(n,m-1\right)\\ & \;+{\tilde{U}}_{\left(i,a\right),\left(i-1,a\right)}{\tilde{p}}_{i-1,a}\left(n,m\right)+{\tilde{U}}_{\left(i,a\right),\left(i,a-1\right)}{\tilde{p}}_{i,a-1}\left(n,m\right)],\\ & \;1\leq i\leq N-1,1\leq a\leq N-i. \end{array}$$

*Submatrices* ${\tilde{U}}_{\left(i,a),(i^{\prime },a^{\prime }\right)}$ *include the transition rates that are associated with transitions from states with* i *symptomatic and* a *asymptomatic to states with* $i^{\prime }$ *symptomatic and* $a^{\prime }$ *asymptomatic cases*.

Proof of Theorem 1 Conditioning on the event that determines the next transition out of the current state $\left(i,a,r,f)\in A_{T}=A-l(0,0\right)$, we get

(18) u˜i,a,r,fp˜i,a,r,f(n,m)=p(f)β(f)Ni,a,rp˜i+1,a,r,f(n−1,m)1−δN−i−a−r,01−δn0+(1−p(f))β(f)Ni,a,rp˜i,a+1,r,f(n,m−1)1−δN−i−a−r,0(1−δm0)+μi(f)p˜i−1,a,r+1,f(n,m)1−δi0+γi(f)p˜i−1,a,r,f(n,m)1−δi0+κa(f)p˜i,a−1,r,f(n,m)1−δa0+∑f′=1f′≠fFuF(f,f′)p˜i,a,r,f′(n,m),

for the set of transient states, while quantities $\left(1-\delta_{kl}\right)$ are used to eliminate probability terms without physical meaning. We note that $\delta_{kl}$ denotes the Kronecker function, which equals one when k=l; otherwise, $\delta_{kl}=0$. Now, formula (18) can be described in matrix form based on formulas ((6), (7), (8), (9), (10), (11), (12), (13), (14), (15), (16), (17)). Employing Equations ((6), (7), (8), (9), (10), (11), (12), (13), (14), (15), (16), (17)) we can compute the joint distribution of the total number of new symptomatic/asymptomatic infections.

The last term of (18) describes the transitions between the EFSs, where $f^{\prime }\neq f\in A_{F}$. The model's parameters are related to the current EFS and are characterized by time‐varying behavior. Finally, the probability vectors ${\tilde{p}}_{0,0}\left(0,0\right)=1_{\left|l\left(0,0\right)\right|}$ and ${\tilde{p}}_{0,0}\left(n,m\right)=0_{\left|l\left(0,0\right)\right|}$ for (n,m)≠(0,0), are associated with the set of absorbing states l(0,0). Apparently, the absence of asymptomatic and symptomatic cases renders the generation of new infections infeasible.

*Remark*. According to Equation (18) we can introduce an alternative expression for the computation of the joint distribution. More specifically, we obtain

(19) $$\begin{array}{cc} \tilde{p}(n,m) & =-{\left({\tilde{U}}_{T}^{*}\right)}^{-1}\left(\left(1-\delta_{n0}\right){\tilde{U}}_{T}^{+}\tilde{p}(n-1,m)\right.\\ & \;\left.+\;\left(1-\delta_{m0}\right){\tilde{U}}_{T}^{-}\tilde{p}(n,m-1)\right), \end{array}$$

for $(n,m)\in ({\mathbb{N}\cup \{0\})}^{2}-\left\{\left(0,0\right)\right\},$ where ${\tilde{U}}_{T}^{*}=({\tilde{u}}_{\left(i,a,r,f),(i^{\prime },a^{\prime },r^{\prime },f^{\prime }\right)}^{*}:\;(i,a,r,f),(i^{\prime },a^{\prime },r^{\prime },f^{\prime })\in A_{T})$, with

(20) $${\tilde{u}}_{\left(i,a,r,f),(i^{\prime },a^{\prime },r^{\prime },f^{\prime }\right)}^{*}=\left\{\begin{array}{ll} -{\tilde{u}}_{i,a,r,f},\; & (i^{\prime },a^{\prime },r^{\prime },f^{\prime })=(i,a,r,f),\\ \gamma_{i}(f),\; & (i^{\prime },a^{\prime },r^{\prime },f^{\prime })=(i-1,a,r,f),\\ \kappa_{a}(f),\; & (i^{\prime },a^{\prime },r^{\prime },f^{\prime })=(i,a-1,r,f),\\ \mu_{i}(f),\; & (i^{\prime },a^{\prime },r^{\prime },f^{\prime })=(i-1,a,r+1,f),\\ u_{F}(f,f^{\prime }),\; & (i^{\prime },a^{\prime },r^{\prime },f^{\prime })=(i,a,r,f^{\prime }),f^{\prime }\neq f,\\ 0,\; & \text{otherwise}, \end{array}\right.$$

while ${\tilde{U}}_{T}^{+}=\left({\tilde{u}}_{\left(i,a,r,f),(i^{\prime },a^{\prime },r^{\prime },f^{\prime }\right)}^{+}:\;(i,a,r,f),(i^{\prime },a^{\prime },r^{\prime },f^{\prime })\in A_{T}\right)$, with

(21) $${\tilde{u}}_{\left(i,a,r,f),(i^{\prime },a^{\prime },r^{\prime },f^{\prime }\right)}^{+}=\left\{\begin{array}{ll} p(f){\left(\frac{\beta (f)}{N}\right)}_{i,a,r}\; & (i^{\prime },a^{\prime },r^{\prime },f^{\prime })=(i+1,a,r,f),\\ 0\; & \text{otherwise}, \end{array}\right.$$

and ${\tilde{U}}_{T}^{-}=\left({\tilde{u}}_{\left(i,a,r,f),(i^{\prime },a^{\prime },r^{\prime },f^{\prime }\right)}^{-}:\;(i,a,r,f),(i^{\prime },a^{\prime },r^{\prime },f^{\prime })\in A_{T}\right)$, with

(22) $$\begin{array}{cc} & {\tilde{u}}_{\left(i,a,r,f),(i^{\prime },a^{\prime },r^{\prime },f^{\prime }\right)}^{-}\\ & \;=\left\{\begin{array}{ll} \left(1-p(f)\right){\left(\frac{\beta (f)}{N}\right)}_{i,a,r}\; & (i^{\prime },a^{\prime },r^{\prime },f^{\prime })=(i,a+1,r,f),\\ 0\; & \text{otherwise}. \end{array}\right. \end{array}$$

The three‐dimensional tensor $\tilde{p}(n,m)$ contains the joint probabilities for every initial state of the set $A_{T}$. Since $\tilde{p}(0,0)$ is known (Equations ((4), (5), (6), (7), (8))), the recursive computation of the joint distribution becomes feasible. An alternative derivation of the $\tilde{p}(0,0)$ vector using generating functions is provided in Appendix A.

Formula (19) seems simpler compared to the matrix equations of Theorem 1. However, it requires the construction and storage of matrices of higher dimension. On the other hand, based on ((4), (5), (6), (7), (8), (9), (10), (11), (12), (13), (14), (15), (16), (17)) we obtain a more efficient computational scheme, while we generate only the necessary matrices for each initial state. Therefore, we recommend the utilization of (19) and ((4), (5), (6), (7), (8), (9), (10), (11), (12), (13), (14), (15), (16), (17)) for small and larger population sizes, respectively. Finally, an algorithmic procedure for the computation of the $\tilde{p}(n,m)$ tensor is presented in Appendix A.

Consequently, the cumulative quantities $N^{S}$ and $N^{A}$ should be interpreted as symptomatic and asymptomatic infection‐event counts. In Variant I, since recovered individuals return to susceptibility, the same individual may contribute more than once to these counts. Therefore, $N^{S}$ and $N^{A}$ are not necessarily bounded by the population size (N).

### 2.3 Distribution of the Maximum Number of Simultaneously Infectious Individuals

In this section, we are interested in investigating extreme tendencies of the system, such as the maximum number of infectious (symptomatic/asymptomatic) cases until the disease's eradication. To facilitate this attempt, we reconstruct the Markov chain of Section 2.2. Namely, we define the absorbing Markov chain 𝒴={Y(t)=(I(t)+A(t),A(t),R(t),F(t)),t≥0}, considering the existence of random external factors that may differentiate the epidemic's parameters.

The new Markov process is associated with the introduction of a new state space, namely B={(i+a,a,r,f):0≤i+a≤N,0≤a≤i+a,0≤r≤N−i−a,1≤f≤F}. The new state space can be partitioned into sublevels, too, where $B={⋃}_{i+a=0}^{N}l^{\prime }(i+a)$ and $l^{\prime }(i+a)={⋃}_{a=0}^{i+a}l^{\prime }(i+a,a).$ Moreover, $l^{\prime }(i+a,a)=\{(i+a,a,r,f):(r,f)\in \{0,\ldots ,N-a-i\}\times \{1,\ldots ,F\}\}$ leading to the absorbing set of states $l^{\prime }(0,0)=\{(0,0,r,f):(r,f)\in \{0,\ldots ,N\}\times \{1,\ldots ,F\}\}$, and the set of transient states ${⋃}_{i+a=1}^{N}l^{\prime }(i+a).$ For the analysis of the maximum infectious burden, we stop the process upon its first entry into the disease‐free set $l^{\prime }(0)$. Accordingly, the transition rates in Equation (23) apply only to states with i+a>0; all transition rates out of disease‐free states are set to zero.

The respective infinitesimal generator matrix $\tilde{Q}=\{{\tilde{q}}_{\left(i+a,a,r,f),(i^{\prime }+a^{\prime },a^{\prime },r^{\prime },f^{\prime }\right)}:(i+a,a,r,f),(i^{\prime }+a^{\prime },a^{\prime },r^{\prime },f^{\prime })\in B\}$, has the form

(23) $$\begin{array}{cc} & {\tilde{q}}_{\left(i+a,a,r,f),(i^{\prime }+a^{\prime },a^{\prime },r^{\prime },f^{\prime }\right)}\\ & \;=\left\{\begin{array}{ll} -{\tilde{q}}_{i+a,a,r,f},\; & (i^{\prime }+a^{\prime },a^{\prime },r^{\prime },f^{\prime })=(i+a,a,r,f),\\ p(f){\left(\frac{\beta (f)}{N}\right)}_{i,a,r},\; & (i^{\prime }+a^{\prime },a^{\prime },r^{\prime },f^{\prime })=(i+a+1,a,r,f),\\ \left(1-p(f)\right){\left(\frac{\beta (f)}{N}\right)}_{i,a,r},\; & (i^{\prime }+a^{\prime },a^{\prime },r^{\prime },f^{\prime })=(i+a+1,a+1,r,f),\\ \gamma_{i}(f),\; & (i^{\prime }+a^{\prime },a^{\prime },r^{\prime },f^{\prime })=(i+a-1,a,r,f),\\ \kappa_{a}(f),\; & (i^{\prime }+a^{\prime },a^{\prime },r^{\prime },f^{\prime })=(i+a-1,a-1,r,f),\\ \mu_{i}(f),\; & (i^{\prime }+a^{\prime },a^{\prime },r^{\prime },f^{\prime })=(i+a-1,a,r+1,f),\\ u_{F}(f,f^{\prime }),\; & (i^{\prime }+a^{\prime },a^{\prime },r^{\prime },f^{\prime })=(i+a,a,r,f^{\prime }),f^{\prime }\neq f,\\ 0,\; & \text{otherwise}, \end{array}\right. \end{array}$$

where ${\tilde{q}}_{i+a,a,r,f}={ũ}_{i,a,r,f}$, for i+a>0, while

(24) $$\tilde{Q}=\left(\begin{array}{ll} 0_{\left|l^{\prime }(0)\right|\times \left|l^{\prime }(0)\right|} & 0_{\left|l^{\prime }(0)\right|\times \left|{⋃}_{i+a=1}^{N}l^{\prime }(i+a)\right|}\\ {\tilde{Q}}_{0} & {\tilde{Q}}_{T} \end{array}\right),$$

where matrix ${\tilde{Q}}_{T}$ contains the transition rates between the transient states and ${\tilde{Q}}_{0}$ connects the transient with the absorbing states. Their forms are

(25) $${\tilde{Q}}_{T}=\left(\begin{array}{lll} \begin{array}{ll} {\tilde{Q}}_{1,1} & {\tilde{Q}}_{1,2}\\ {\tilde{Q}}_{2,1} & {\tilde{Q}}_{2,2} \end{array} & \cdots & \begin{array}{l} 0\\ 0 \end{array}\\ \vdots & \ddots & \vdots \\ \begin{array}{ll} 0\; & 0 \end{array} & \cdots & {\tilde{Q}}_{N,N} \end{array}\right),$$

and

(26) $${\tilde{Q}}_{0}=\left(\begin{array}{l} {\tilde{Q}}_{1,0}\\ 0_{\left|{⋃}_{i+a=2}^{N}l^{\prime }(i+a)\right|\times |l^{\prime }(0)|} \end{array}\right).$$

Matrices ${\tilde{Q}}_{i,j}$ contain the transition rates from level $l^{\prime }(i)$ (i infectious cases) to $l^{\prime }(j)$ (j infectious cases).

Since we are interested in the maximum number of simultaneously infectious cases until the disease's extinction, we define the random variable

$$\begin{array}{cc} \tau & =\inf\{t\geq 0:\;(I(t)+A(t),A(t),R(t),F(t))=(0,0,r,f),\;\\ & \;\;0\leq r\leq N,1\leq f\leq F\}, \end{array}$$

which represents the duration of the epidemic. Next, we define the random variable $\xi =\max_{t\in [0,\tau )}(I(t)+A(t))$ that denotes the maximum number of simultaneously infectious cases. Our objective is the computation of the cumulative distribution function of the maximum number of simultaneously infectious cases,

(27) $$F_{i+a,a,r,f}(x)=\left\{\begin{array}{ll} 0,\; & x<1,\\ P\left(\xi \leq x|Y(0)=(i+a,a,r,f)\right),\; & 1\leq x<N,\\ 1,\; & x\geq N. \end{array}\right.$$

It should be noted that the subscripts of the $F_{i+a,a,r,f}(x)$ function denote the selected initial state.

Theorem 2 *The vector of cumulative distribution functions which are associated with the maximum number of simultaneously infectious* (*symptomatic/asymptomatic*) *cases, for* y=1,…,N, *can be computed according to*

(28) $$\begin{array}{cc} F(y) & =-{\tilde{Q}}_{y}^{-1}\left(\left(\delta_{i+a,1}\delta_{a,1}\kappa_{1}(f)+{\delta_{i+a,1}\delta }_{a,0}\left(\mu_{1}(f)+\gamma_{1}(f)\right)\right)\right.\\ & \;\times \left.1_{\left|{⋃}_{i+a=1}^{y}l^{\prime }(i+a)\right|}\right)\;. \end{array}$$

*The column vector* ${\tilde{q}}_{0}(y)=\left(\delta_{i+a,1}\delta_{a,1}\kappa_{1}(f)+\delta_{i+a,1}\delta_{a,0}\left(\mu_{1}(f)+\right.\right.\left.\left.\gamma_{1}(f)\right)\right)1_{\left|{⋃}_{i+a=1}^{y}l^{\prime }(i+a)\right|}$ *represents the transition rates from the set of transient states* ${⋃}_{i+a=1}^{y}l^{\prime }(i+a)$ *to the absorbing set* $l^{\prime }(0)$. *Matrix* ${\tilde{Q}}_{y}$ *is a submatrix of* $\tilde{Q}$ *containing the transition rates between the states of the set* ${⋃}_{i+a=1}^{y}l^{\prime }(i+a)$.

For a fixed y=1,…,N, we notice that $F_{i+a,a,r,f}(y)$ equals the conditional probability that starting from Y(0)=(I(0)+A(0),A(0),R(0),F(0))=(i+a,a,r,f), the Markov chain 𝒴 enters the set of states $l^{\prime }(0)$ without visiting the states of $l^{\prime }(y+1).$ The absorption of the Markov process which derives from the eradication of the disease is inevitable, since the state space B contains a unique closed absorbing set. After removing the rows and columns of matrix ${\tilde{Q}}_{T}$ that correspond to states where I(t)+A(t)>y, we ensure that during the outbreak, the maximum number of simultaneously infectious subjects is at most y. According to the above, the computation of the vector of cumulative probabilities F(y) becomes feasible.

Since we are interested in estimating the probability of I(t)+A(t)≤y, for a fixed y=1,…,N, we introduce the auxiliary Markov chain 𝒴(y)={Y(y,t),t≥0}. This chain is defined in the state space $B(y)=l^{\prime }(0)⋃\left({⋃}_{i+a=1}^{y}l^{\prime }(i+a)\right)⋃l^{\prime }(y+1)$, where $l^{\prime }(0)$ and $l^{\prime }(y+1)$ represent sets of absorbing states. For simplicity, these sets can be lumped into two absorbing states, leading to $B(y)=\{0\}⋃\left({⋃}_{i+a=1}^{y}l^{\prime }(i+a)\right)⋃\{y+1\}$.

Finally, the corresponding generator matrix becomes [46, 47]

(29) $$\tilde{Q}(y)=\left(\begin{array}{lll} 0 & 0_{\left|{⋃}_{i+a=1}^{y}l^{\prime }(i+a)\right|}^{T} & 0\\ {\tilde{q}}_{0}(y) & {\tilde{Q}}_{y} & {\tilde{q}}_{y+1}(y)\\ 0 & 0_{\left|{⋃}_{i+a=1}^{y}l^{\prime }(i+a)\right|}^{T} & 0 \end{array}\right),$$

where ${\tilde{q}}_{0}(y)$ and ${\tilde{q}}_{y+1}(y)$ are column vectors of order ${⋃}_{i+a=1}^{y}l^{\prime }(i+a)$. Square matrix ${\tilde{Q}}_{y}$ is of the same dimension, while it includes the transition rates between the transient states of the auxiliary Markov chain.

The column vector ${\tilde{q}}_{0}(y)=\left(\delta_{i+a,1}\delta_{a,1}\kappa_{1}(f)+\delta_{i+a,1}\delta_{a,0}\left(\mu_{1}(f)+\right.\right.\left.\left.\gamma_{1}(f)\right)\right)1_{\left|{⋃}_{i+a=1}^{y}l^{\prime }(i+a)\right|}$, contains the transition rates from the set ${⋃}_{i+a=1}^{y}l^{\prime }(i+a)$ to the absorbing set {0}. The values of parameters $\mu_{1}(f)$, $\gamma_{1}(f)$ and $\kappa_{1}(f)$ vary according to the EFS associated with each initial state in ${⋃}_{i+a=1}^{y}l^{\prime }(i+a)$. The Kronecker delta function $\delta_{a,1}$ is used to clarify that the $\kappa_{1}(f)$ terms are included in the rows of ${\tilde{q}}_{0}(y)$ corresponding to the presence of one asymptomatic case. Based on the above, the vector of conditional probabilities can be obtained using (28), where $F(y)=\;-{\tilde{Q}}_{y}^{-1}{\tilde{q}}_{0}(y)$, which signifies the probability that 𝒴(y) enters {0}, without visiting the states of the set {y+1}. Finally,

(30) $${\tilde{q}}_{y+1}(y)=\;-{\tilde{Q}}_{y}1_{\left|{⋃}_{i+a=1}^{y}l^{\prime }(i+a)\right|}-{\tilde{q}}_{0}(y),$$

as the rows of $\tilde{Q}(y)$ sum to zero.

Equation (28) is also intuitively consistent, as for a fixed y=1,…,N, matrix $-{\tilde{Q}}_{y}^{-1}$ provides the expected time that the process 𝒴(y), spends in the transient states times a column vector that contains the transition rates to the set $l^{\prime }(0)$. Consequently, the process does not visit any states where the number of simultaneously infectious cases exceeds the selected threshold y.

It should be noted that symptomatic and asymptomatic infectious individuals make the same per capita contribution to transmission. Thus, the infection rate per external factor, β(f),f=1,…,F, is interpreted as a common effective transmission rate for both infectious classes. This simplifying assumption keeps the finite‐population distributional analysis tractable and is also used in the H1N1 illustration of Section 3.3, because separate reliable estimates of symptomatic and asymptomatic transmissibility are not available.

## 3 Results

In this section, we examine how different sets of parameters influence the behavior of the stochastic SuASR framework under random external factors and the analyzed descriptors. By systematically exploring multiple parameter sets under diverse initial and environmental factors, we aim to derive analytical insights into the stochastic behavior of epidemic spread. These findings can inform public health decision‐making by enabling the formulation of more targeted and temporally responsive intervention strategies.

For the remainder of this section, we employ relatively small and moderate population sizes for illustrative purposes. Thus, we use formula (19) to compute the joint distribution of new symptomatic and asymptomatic infections. However, the use of moderate population sizes is appropriate for several localized epidemic contexts, including outbreaks in hospital wards, schools, military training camps, and other closed or semi‐closed communities, where stochastic effects remain non‐negligible and finite‐population modeling is justified [25, 48]. It is important to stress that N does not necessarily represent the population of an entire geographical region, but rather the subset of individuals who are realistically involved in the transmission process. Under this interpretation, the initial number of susceptible individuals becomes an epidemiologically meaningful parameter that may itself require estimation, as discussed in [12, 49, 50, 51]. Therefore, moderate population sizes may also be suitable when analyzing epidemic spread on a broader scale.

Sections 3.1 and 3.2 are based on Variant I and are intended as methodological sensitivity analyses. For the numerical evaluation of the joint infection‐count distributions in these sections, we use the truncation limits $n_{\max}=m_{\max}=150$, retaining counts 0≤n,m≤150. Section 3.3 uses Variant II for the H1N1 influenza application, where reinfections are assumed negligible over the study period.

### 3.1 The Impact of the System's Time‐Varying Parameters

In this section, we investigate the impact of model parameters on the joint distribution of the number of symptomatic/asymptomatic infections, along with the maximum number of simultaneously infectious cases, using the model variant presented in Section 2.1.1. We assume that the state space of external random effects consists of three states, that is, f∈{1,2,3}. For illustrative purposes, we consider a population size of N=20, and symptomatic ratio p=0.6. Higher population sizes can be examined in a similar manner. Moreover, we select β∈{(0.2,0.7,1.2),(0.6,1.1,1.6)}, γ=κ∈{(0.3,0.15,0.05),(0.5,0.35,0.2)}, and μ∈{(0.02,0.05,0.1),(0.05,0.1,0.15)}. These parametric triplets represent a progression towards a more severe disease, potentially attributed to the onset of variants. We choose $u_{F}(1,2)=\frac{1}{90}$, $u_{F}(2,3)=\frac{1}{60}$, and $u_{F}(f,f^{\prime })=\;0$, otherwise. The initial state is set as (i,a,r,f)=(5,5,5,1). Based on the above, the process remains in the first EFS on average for 90 time units, transitions to the second EFS where it stays for an average of 60 time units and then persists indefinitely in the third EFS.

The resulting joint probability distributions are illustrated in Figure 1. The omitted probability mass is less than $10^{-6}$ in all instances. We observe a higher incidence of symptomatic infections, as anticipated given p=0.6. The configuration β=(0.6,1.1,1.6), γ=κ=(0.3,0.15,0.05), and μ=(0.02,0.05,0.1) corresponds to a more severe manifestation of the disease. Under this scenario, a bimodal distribution emerges with expected numbers of $E(N^{S})=61.85$ and $E(N^{A})=41.25$. This pattern results from the intensified infection rates, which lead to a rapid depletion of susceptible individuals. At the same time, the relatively lower recovery and mortality rates prolong the time that individuals remain infectious, thereby facilitating the continued generation of new cases.

**FIGURE 1:**
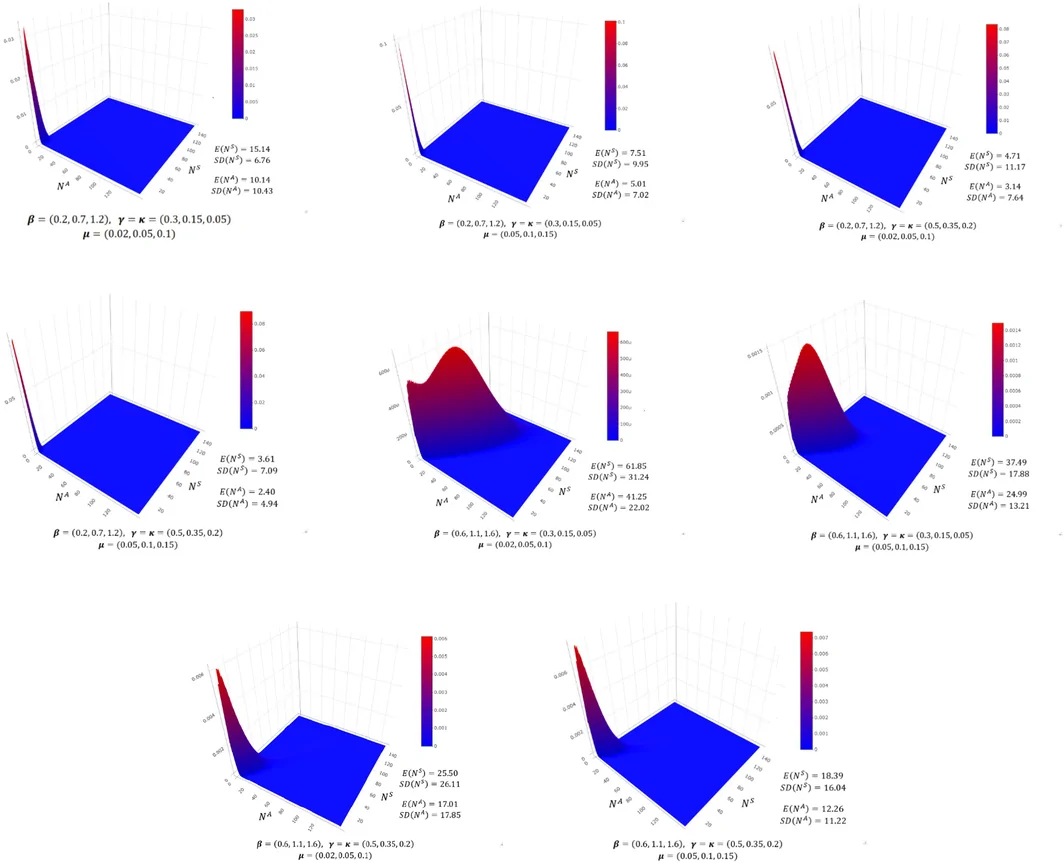
Joint probability distributions of the total number of new symptomatic $\left(N^{S}\right)$ and asymptomatic $\left(N^{A}\right)$ infections. In each graph we include the expected value and standard deviation of the respective marginal distribution.

Conversely, when considering parameter combinations that hinder the transmission of the disease, characterized by higher recovery and mortality and lower transmission rates, we observe unimodal joint distributions with a peak at zero. Hence, it becomes evident that higher expected numbers of infections are associated with greaterβ and lower γ, κ, and μ rates. This trend results in a system with a higher prevalence of infectious cases, serving as the driving force behind the observed phenomenon.

Now, we choose the initial state (i+a,a,r,f)=(1,1,0,1), which corresponds to the presence of only one asymptomatic case. Unimodal decreasing forms with a mode at 1 are observed when the impact of the exit rates from the infectious phase surpasses the virus's transmission (Figure 2) which can lead to the eradication of the disease before the number of simultaneously infectious individuals exceeds the initial count. In scenarios more favorable to disease transmission, a second mode appears, resulting in higher expected values. This pattern is similarly reflected in the variance, where greater variability (and thus greater uncertainty) corresponds to a wider range of trajectories for the stochastic process.

**FIGURE 2:**
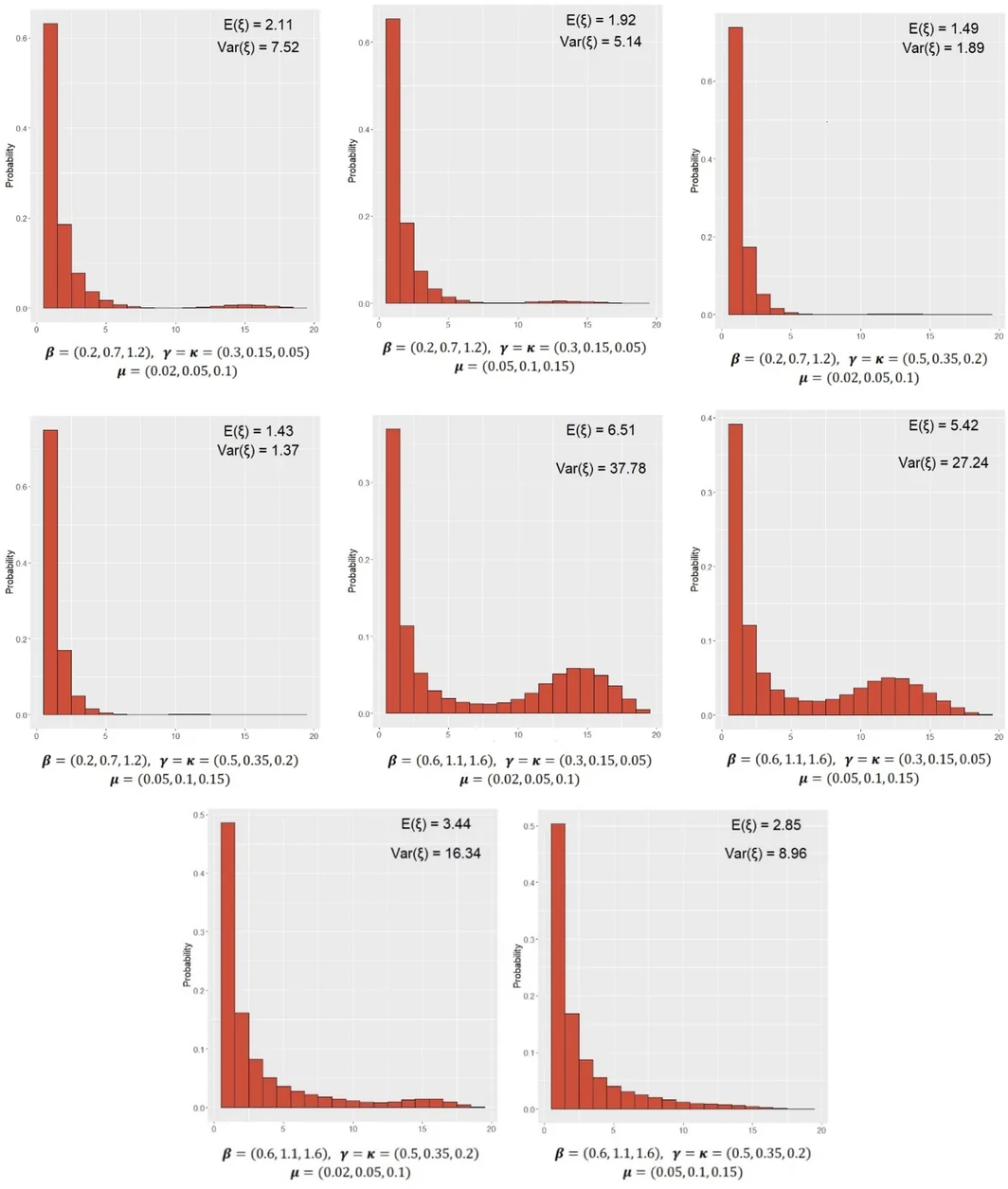
Probability distributions of the maximum number of simultaneously infectious cases (ξ) along with the respective expected value and variance.

To validate the numerical implementation of the proposed matrix‐recursive formulas, we also considered two additional small‐population scenarios and compared the exact values obtained from our method with direct Gillespie simulations of the CTMC. In both scenarios, we set N=20, F=3, and the initial state equal to (I(0),A(0),R(0),F(0))=(1,1,5,1). In the first case, we use β=(1.0,1.5,0.5), p=0.4, κ=(0.3,0.3,0.3), γ=(0.2,0.2,0.2), μ=(0.15,0.05,0.01), with EFS transition rates $u_{F}(1,2)=\;u_{F}(1,3)=\frac{1}{10}$, $u_{F}(2,1)=\;u_{F}(2,3)=\frac{1}{15}$, $u_{F}(3,1)=\;u_{F}(3,2)=\frac{1}{25}$ and $u_{F}(f,f^{\prime })=\;0$, otherwise. In the second case, we use β=(1.5,2.0,0.8), p=0.6, κ=(0.25,0.30,0.35), γ=(0.15,0.20,0.25), μ=(0.10,0.05,0.02), with EFS transition rates $u_{F}(1,2)=\;u_{F}(1,3)=\frac{1}{5}$, $u_{F}(2,1)=\;u_{F}(2,3)=\frac{1}{20}$, and $u_{F}(3,1)=\;u_{F}(3,2)=\frac{1}{30}$.

This comparison is not intended to show that the matrix‐recursive method is merely an efficient approximation. On the contrary, the formulas developed in Sections 2.2 and 2.3 provide exact computations of the joint infection‐count distribution, the maximum infectious‐burden distribution, and their corresponding moments. The omitted probability mass is less than $10^{-4}$ in both cases. The Gillespie algorithm, by contrast, provides simulation‐based estimates of the same quantities. Therefore, the agreement between the two approaches should be interpreted as a validation of the computational implementation of the proposed exact formulas. According to Table 1, in the two cases considered, the exact and Gillespie‐based values were very close for the expected symptomatic and asymptomatic counts, as well as for the expected maximum infectious burden and standard deviations, supporting the numerical correctness of the implementation.

**TABLE 1:** Validation of the exact computations against Gillespie simulations.

Case	Quantity	Exact computation	Gillespie estimate	Absolute difference
1	$E(N^{S})$	16.202	16.156	0.046
1	$E(N^{A})$	24.302	24.311	0.009
1	$SD(N^{S})$	13.751	13.641	0.110
1	$SD\left(N^{A}\right)$	21.117	21.138	0.021
1	E(ξ)	8.833	8.865	0.032
1	SD(ξ)	4.132	4.159	0.027
2	$E(N^{S})$	35.996	35.979	0.017
2	$E(N^{A})$	23.997	24.039	0.042
2	$SD(N^{S})$	20.527	20.263	0.264
2	$SD\left(N^{A}\right)$	14.610	14.453	0.157
2	E(ξ)	11.341	11.332	0.009
2	SD(ξ)	3.423	3.421	0.002

### 3.2 Exploring the Influence of Random External Factors

After conducting the sensitivity analysis outlined above, which illustrates how the parametric space influences disease severity, we proceed to examine the impact of transition rates between the defined EFSs, using the model variant presented in Section 2.1.1. We present three distinct scenarios, each representing a unique transition pattern, with the aim of uncovering the underlying dynamics of the stochastic system. It is important to note that, for each scenario, the remaining parameters have been carefully selected to enable a focused examination of the intended patterns.

*Scenario A*:

First, we select (i,a,r,f)=(3,3,1,1) as initial state which is associated with a total of six currently infectious individuals. For the three cases of this scenario, we select:

(A) N=30, μ=0.05, p=0.7, β=0.5, κ=0.5, γ=0.3, and $u_{F}\left(f,f^{\prime }\right)=0$, for $f,f^{\prime }\in \{1,2,3\}$,
(B) N=30, μ=0.05,p=0.7, β=(0.5,1,2), κ=(0.5,0.2,0.1), γ=(0.3,0.1,0.05), while $u_{F}(1,2)=\frac{1}{15}$, $u_{F}(2,3)=\frac{1}{5}$, and $u_{F}(f,f^{\prime })=0$, otherwise,
(C) N=30, μ=0.05,p=0.7, β=(0.5,1,2), κ=(0.5,0.2,0.1), γ=(0.3,0.1,0.05), while $u_{F}(1,2)=\frac{1}{5}$, $u_{F}(2,3)=\frac{1}{15}$, and $u_{F}(f,f^{\prime })=0$, otherwise.

The first case represents a straightforward scenario in which the corresponding CTMC can be modeled using Equation (2), as there are no transition rates between the EFSs. In cases (B) and (C), we deliberately use the same parameter combinations, which are associated with increasing disease severity. This is reflected in the rising infection trends and the contrasting behavior of recovery rates. It is also worth noting that the stochastic process cannot exit the third EFS. The main distinction between these two cases is that, based on the chosen transition rates, the process remains in the first (less severe) EFS for a longer period in case (B).

Figures 3 and 4 present the joint probability distribution of symptomatic/asymptomatic infections (with omitted probability mass less than $10^{-5}$ in all three cases), as well as the probability distribution of the maximum number of infectious individuals, along with the corresponding expected values and variances or standard deviations. According to Figure 3, case (C) yields expected values of 55.47 symptomatic and 23.77 asymptomatic infections, displaying a bimodal distribution with a more pronounced second peak compared to the milder case (B). The higher expected number of symptomatic infections, relative to asymptomatic cases, is consistent with the chosen symptomatic ratio p=0.7. In case (A), which represents the least severe epidemic scenario, the second peak of the distribution disappears, as the infection rate remains consistently low throughout the duration of the outbreak.

**FIGURE 3:**
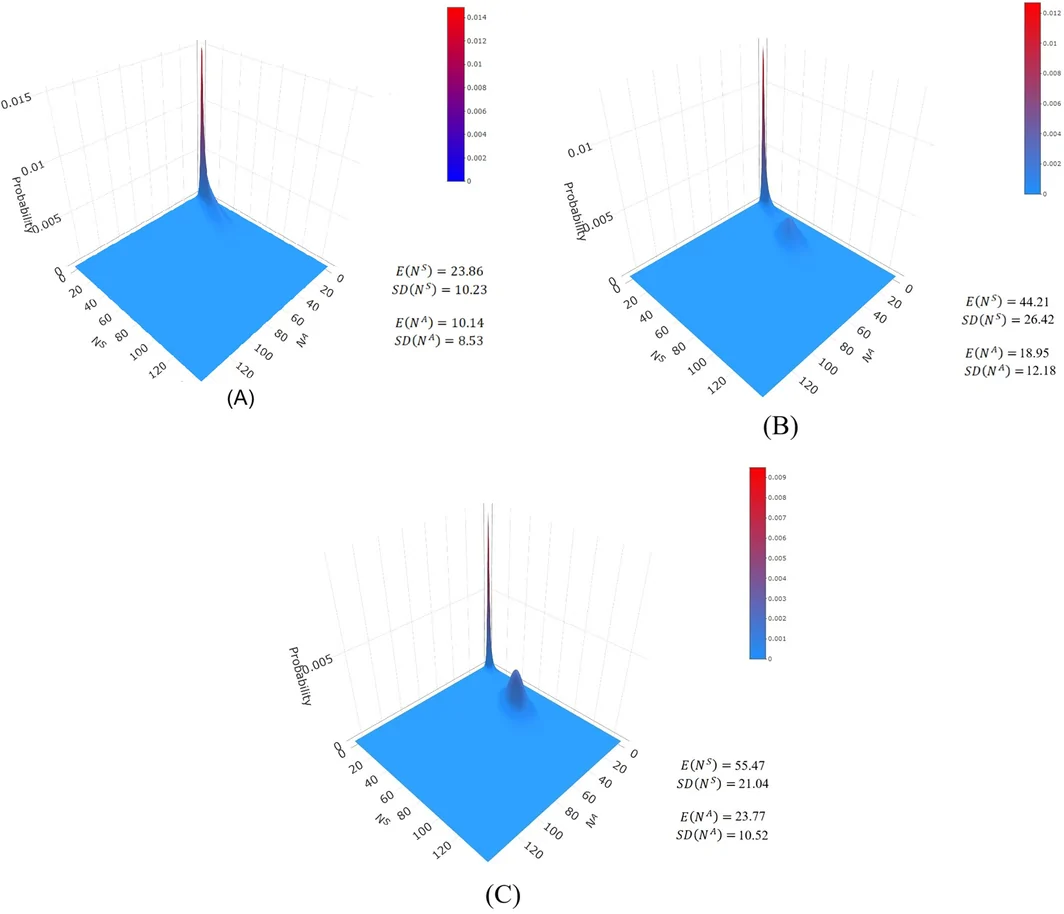
Joint probability distributions of the total number of new symptomatic $\left(N^{S}\right)$ and asymptomatic $\left(N^{A}\right)$ infections for the three cases of scenario A. In each graph we include the expected value and standard deviation of the marginal distributions of symptomatic and asymptomatic infections.

**FIGURE 4:**
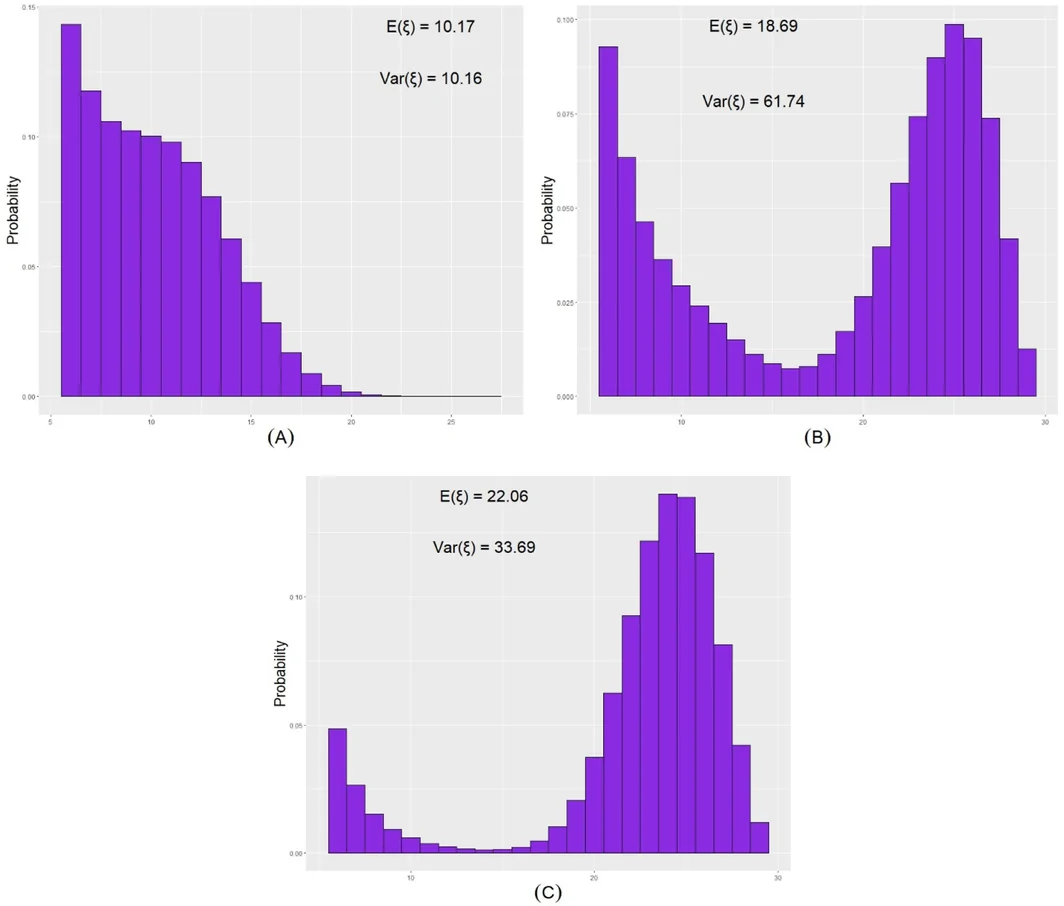
Probability distributions of the maximum number of simultaneously infectious cases (ξ) along with the respective expected value and variance corresponding to scenario A.

This behavior is also evident in Figure 4, where the highest expected value corresponds to case (C). As for the joint distribution, in case (A), the bimodal distributions observed in cases (B) and (C) are replaced by a unimodal distribution with a peak at six infectious individuals. It should be noted that, given the chosen values (i,a,r,f)=(3,3,1,1), the corresponding initial state for the modified process described in Section 2.3 is (i+a,a,r,f)=(6,3,1,1). As a result, the value six represents the minimum possible value of this stochastic descriptor. Finally, the highest uncertainty is observed in case (B), with Var(ξ)=61.74. Based on the shape of the distribution, significant probability mass is concentrated at both low and high values of infectious individuals. This indicates the possibility of both early epidemic eradication and trajectories where the number of infectious individuals approaches the maximum capacity.

*Scenario B*:

In this scenario, we examine the impact of the transition rates among the EFSs while maintaining a constant population size (N) and a fixed symptomatic ratio (p). The transmission rate decreases when transitioning from EFS 1 to EFS 2 and then increases again when moving from EFS 2 to EFS 3, though it remains lower than the initial rate. This behavior may result from the implementation of a public health measure, such as a lockdown, where the infectious force rises again after the measure is lifted but does not return to its original level. This may be due to partial resistance developed within the population during the intervention period. Another contributing factor could be the vaccination of the susceptible population with an imperfect vaccine. While the vaccine initially reduces infectiousness, a period of increased transmission may follow once new vaccinations stop and herd immunity weakens due to limited protection against the virus. Finally, it is also instructive to compare this scenario with the standard case characterized by constant epidemic parameters.

Aiming to satisfy the aforementioned characteristics, we consider the following three cases:

(A) N=30, p=0.5, β=2, κ=0.3, γ=0.2, μ=0.25, and $u_{F}(f,f^{\prime })=0$, for $f\neq f^{\prime }\in \left\{1,\;2,\;3\right\}$,
(B) N=30, p=0.5, β=(2,0.5,1), γ=(0.2,0.3,0.4), κ=(0.3,0.4,0.5), μ=(0.25,0.15,0.05), while $u_{F}(1,2)=\frac{1}{5}$, $u_{F}(2,3)=\frac{1}{5}$, and $u_{F}(f,f^{\prime })=0$, otherwise,
(C) N=30, p=0.5, β=(2,0.5,1), γ=(0.2,0.3,0.4), κ=(0.3,0.4,0.5), μ=(0.25,0.15,0.05), while $u_{F}(1,2)=\frac{1}{5}$, $u_{F}(2,3)=\frac{1}{15}$, and $u_{F}(f,f^{\prime })=0$, otherwise.

The only difference between cases (B) and (C) is the transition rate from the second to the third EFS; consequently, the process remains in the second EFS for a longer period in case (C). We select the initial state (i,a,r,f)=(2,2,8,1), comprising two symptomatic, two asymptomatic, and eight removed cases, and begin our analysis from the first EFS. The omitted mass for the computation of the infection‐count distribution is less than $10^{-5}$ in all three cases.

Given that p=0.5, the infected cases are evenly distributed between the symptomatic and asymptomatic compartments. As a result, the joint distributions are nearly symmetric with respect to the diagonal y=x. Case (A) is associated with the highest total number of new symptomatic and asymptomatic infections, with expected values of 31.53 (Figure 5). This outcome is attributed to the constant and high infection rate characterizing the first EFS. Since no transitions between EFSs are permitted, the process remains at the maximum level of infectiousness throughout the entire period. As expected, case (C) results in the lowest number of infections, as the stochastic process stays in the second EFS (associated with a lower transmission rate) for the longest period.

**FIGURE 5:**
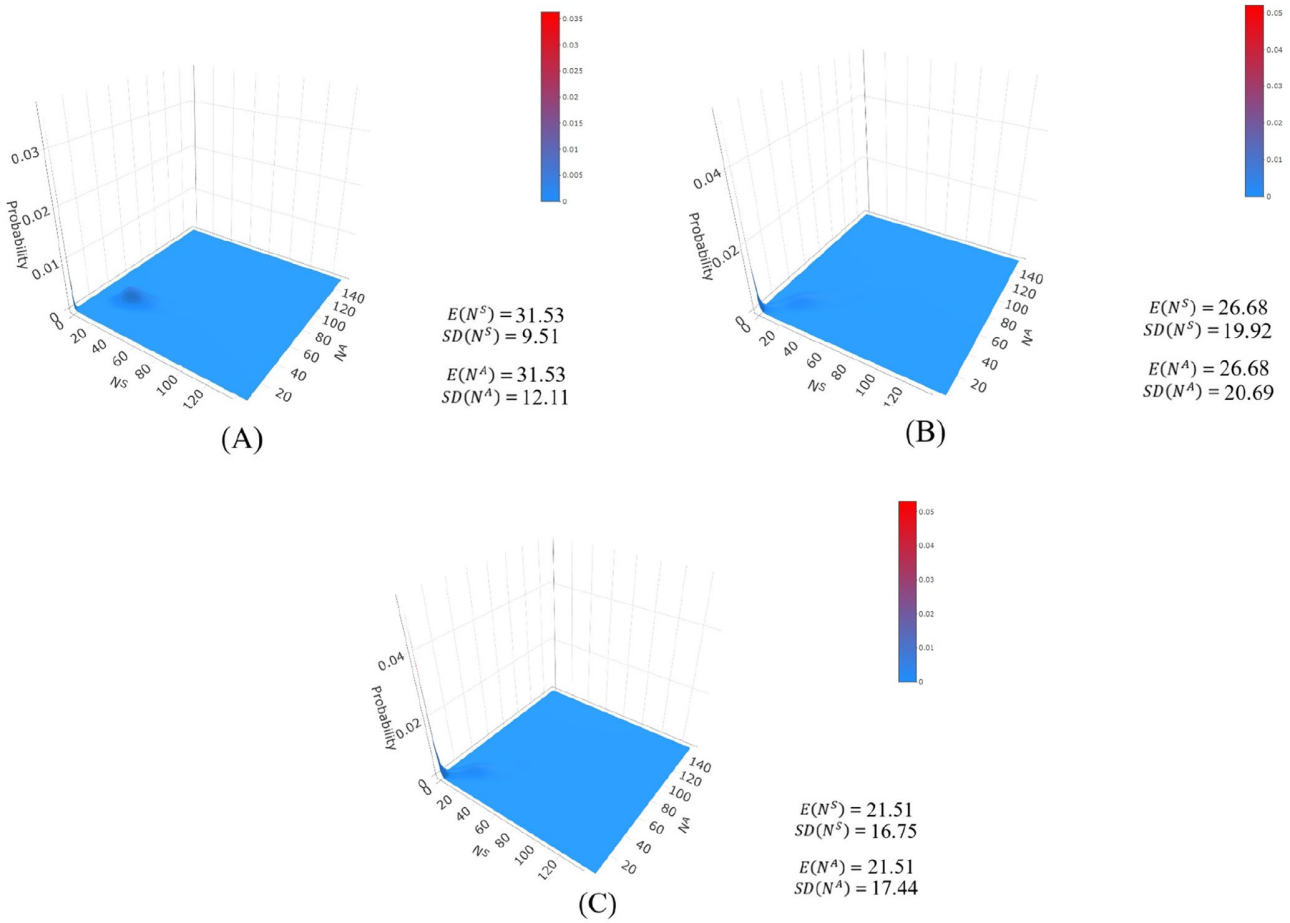
Joint probability distributions of the total number of new symptomatic $\left(N^{S}\right)$ and asymptomatic $\left(N^{A}\right)$ infections for the three cases of scenario B. In each graph we include the expected value and standard deviation of the marginal distributions of symptomatic and asymptomatic infections.

When considering the maximum number of simultaneously infectious cases, case (A) yields the highest expected value, reflecting the persistently high transmission rate in the first EFS. Cases (B) and (C) both reduce the total and peak epidemic burden relative to case (A). According to Figures 5 and 6, case (B) produces slightly higher expected new infections and a slightly higher expected peak than case (C). This is consistent with the shorter stay in the low‐transmission second EFS, which allows the process to reach the third EFS earlier.

**FIGURE 6:**
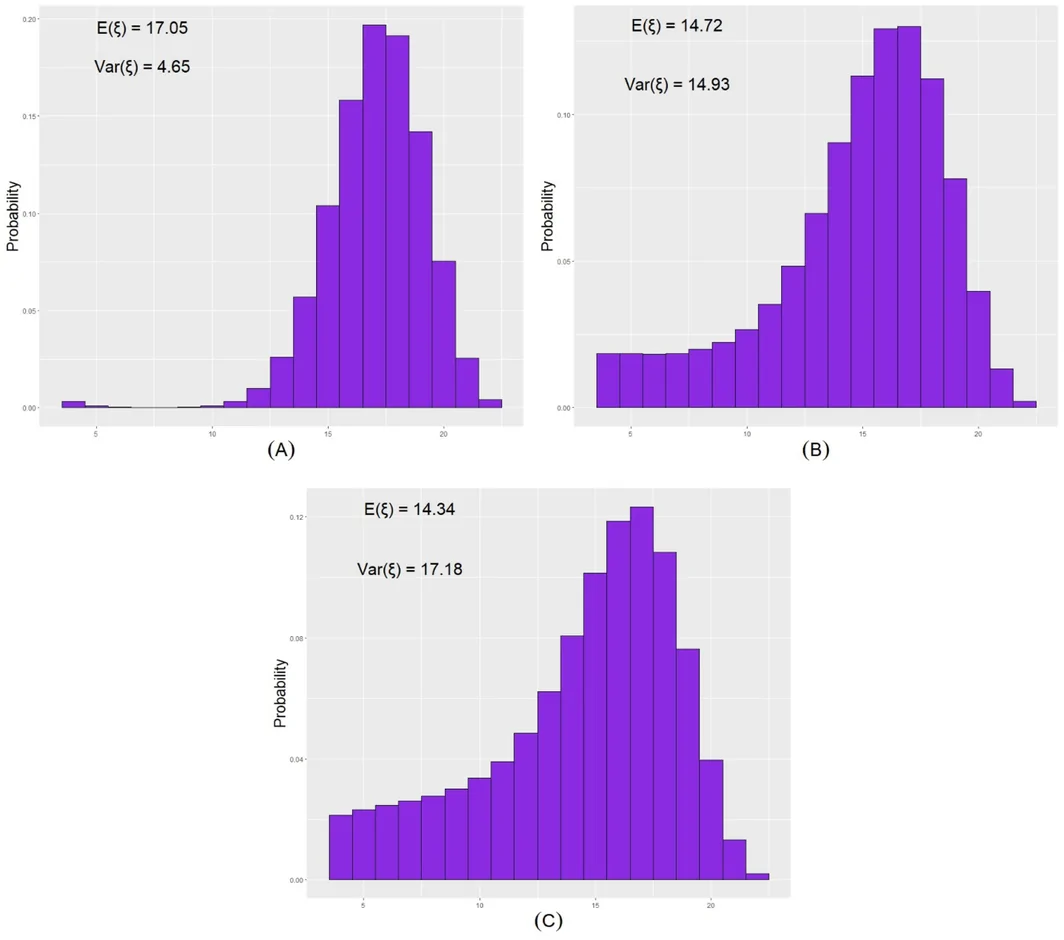
Probability distributions of the maximum number of simultaneously infectious cases(ξ) along with the respective expected value and variance corresponding to scenario B.

All three diagrams originate from an initial state with four infectious cases. The initial state for the modified Markov chain is set to (i+a,a,r,f)=(4,2,8,1), meaning that the value 4 represents the minimum value for the examined stochastic descriptor.

*Scenario C*:

In all cases of scenario C, we present a cyclical pattern, while we vary the expected durations of the process to each EFS. The same initial state as in scenario A is selected here, too. We deem that the external factors impact only the transmission and mortality rates. More specifically, we set:

(A) N=20, p=0.4, γ=0.2, κ=0.3, β=(1,2,0.5), μ=(0.2,0.15,0.1), while $u_{F}(1,2)=u_{F}(1,3)=\frac{1}{5}$, $u_{F}(2,3)=u_{F}(2,1)=\frac{1}{15}$, $u_{F}(3,1)=u_{F}(3,2)=\frac{1}{25}$, and $u_{F}(f,f^{\prime })=0$, otherwise,
(B) N=20, p=0.4, γ=0.2, κ=0.3, β=(1,2,0.5), μ=(0.2,0.15,0.1), while $u_{F}(1,2)=u_{F}(1,3)=\frac{1}{25}$, $u_{F}(2,3)=u_{F}(2,1)=\frac{1}{5}$, $u_{F}(3,1)=u_{F}(3,2)=\frac{1}{15}$, and $u_{F}(f,f^{\prime })=0$, otherwise,
(C) N=20, p=0.4, γ=0.2, κ=0.3, β=(1,2,0.5), μ=(0.2,0.15,0.1), while $u_{F}(1,2)=u_{F}(1,3)=\frac{1}{15}$, $u_{F}(2,3)=u_{F}(2,1)=\frac{1}{25}$, $u_{F}(3,1)=u_{F}(3,2)=\frac{1}{5}$, and $u_{F}(f,f^{\prime })=0$, otherwise.

It should be noted that, in all three cases, a higher number of asymptomatic infections is observed, which is consistent with the selected symptomatic ratio. The omitted probability mass in the cases of this scenario is less than $10^{-7}$.

In scenario C, the transition structure is not directed as in scenarios A and B. Since each EFS may have more than one outgoing transition, the expected holding time in each EFS is computed as the reciprocal of the total exit rate from that state, rather than from a single transition rate.

According to Figures 7 and 8, case (B) represents the mildest instance of an epidemic when considering both descriptors, with 19.26 symptomatic and 28.88 asymptomatic individuals, and an expected maximum of 13.01 infectious individuals. The highest expected total number of infections until disease extinction occurs in case (C), with 59.92 infections. This case reflects increased epidemic severity, as based on the selected expected durations for the EFSs, the process spends more time in the first two, and especially in the second EFS, where the infection rate is two to four times higher than in the other EFSs. In contrast, in case (B), the process spends the least amount of time in the second EFS, resulting in a reduced number of total infections.

**FIGURE 7:**
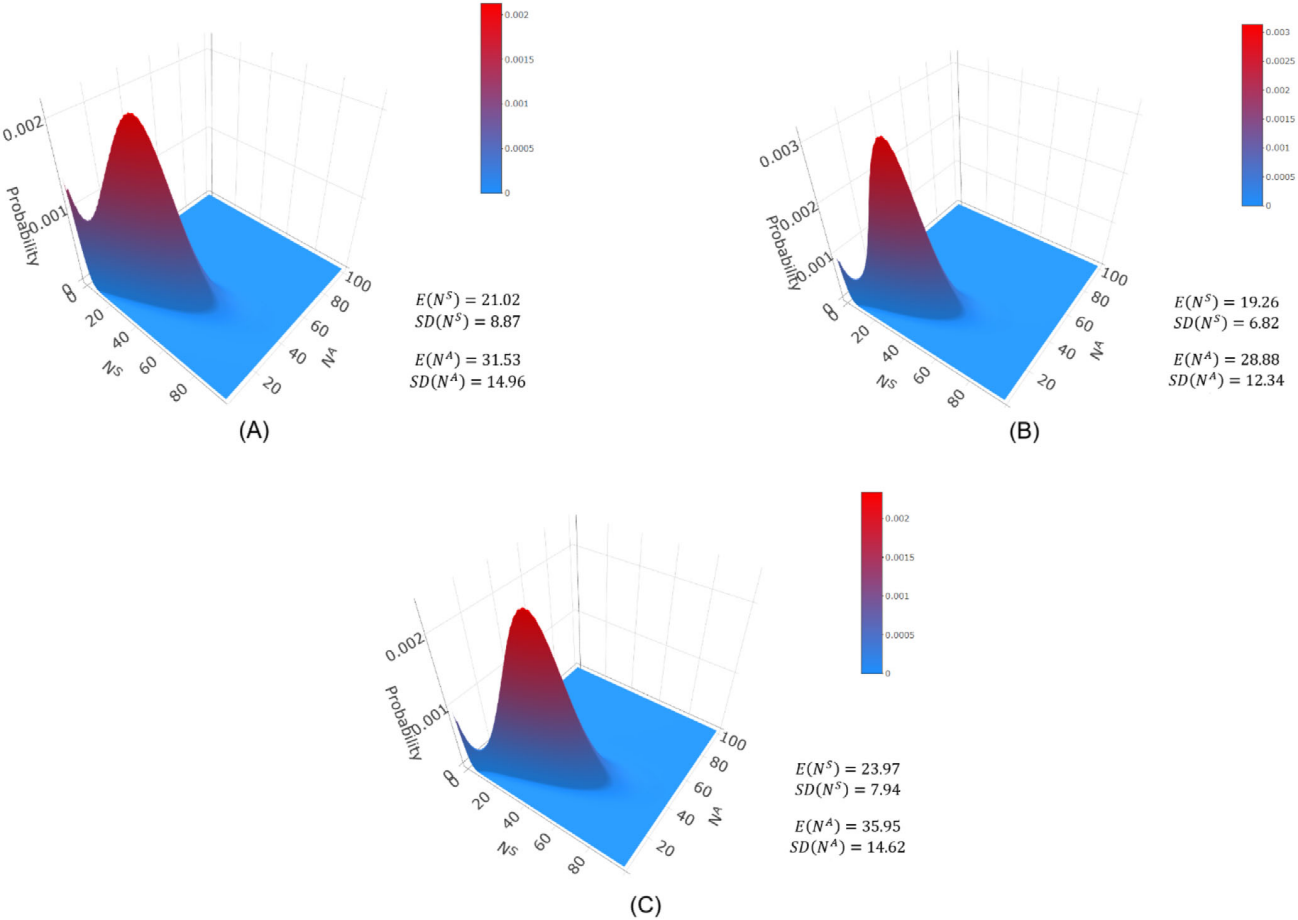
Joint probability distributions of the total number of new symptomatic $\left(N^{S}\right)$ and asymptomatic $\left(N^{A}\right)$ infections for the three cases of scenario C. In each graph we include the expected value and standard deviation of the marginal distributions of symptomatic and asymptomatic infections.

**FIGURE 8:**
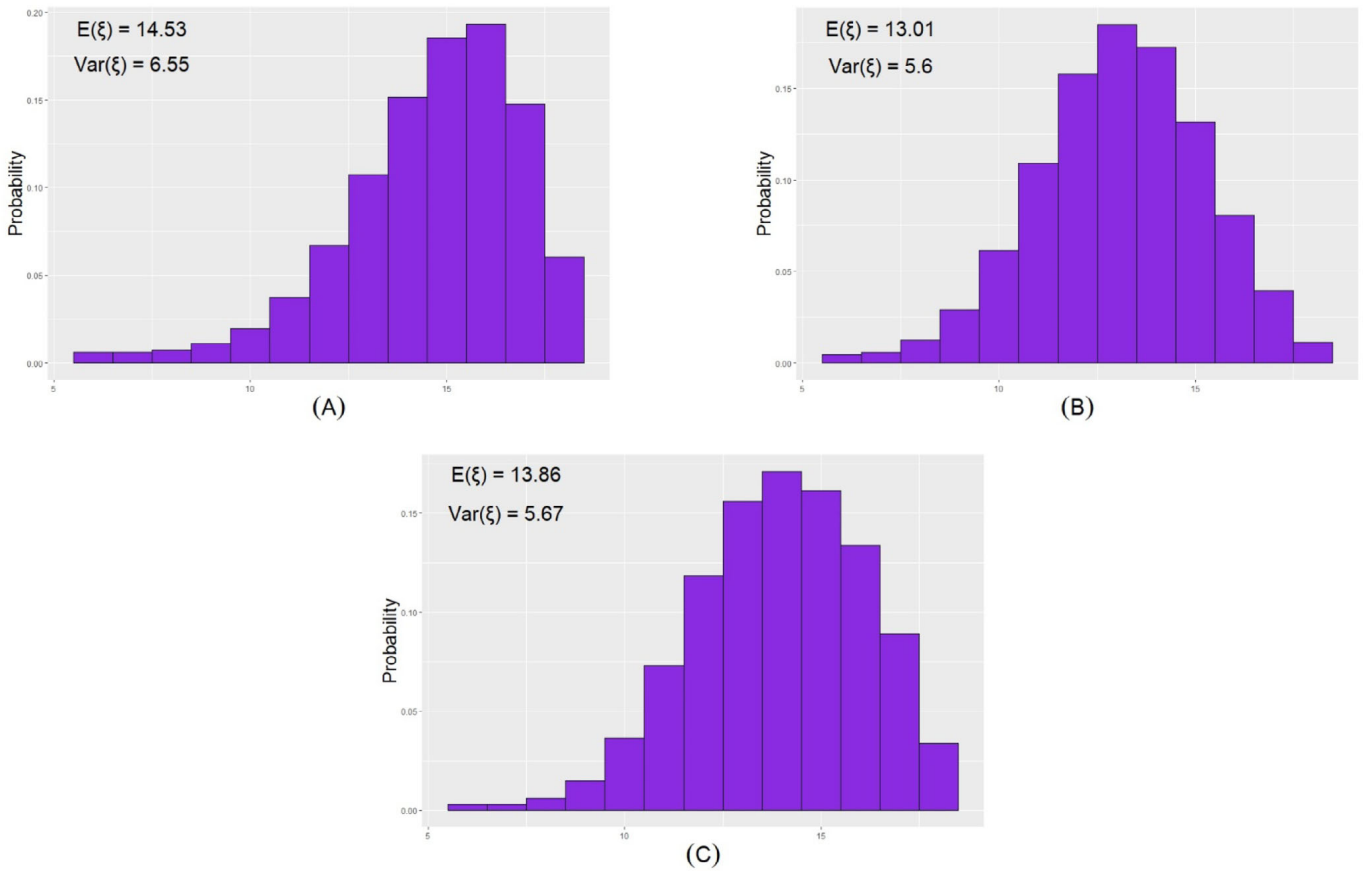
Probability distributions of the maximum number of simultaneously infectious cases (ξ) along with the respective expected value and variance corresponding to scenario C.

The case associated with the highest total number of infections does not coincide with the one exhibiting the highest infection peak. This observation underscores the importance of analyzing both measures to gain a more comprehensive understanding of epidemic dynamics. Finally, the joint distributions of all three cases exhibit bimodal forms, with the highest variance observed in case (A). This can be explained by the shape of the distribution, where significant probability mass is concentrated at both zero and higher numbers of total infections. This outcome corresponds to epidemic trajectories that may either die out quickly or persist for a considerable period.

### 3.3 Application to the 2009 H1N1 Influenza Pandemic

H1N1 influenza pandemic of 2009 or Swine‐Flu began its spread from Mexico and its transmission is believed to follow a pattern similar to that of seasonal flu [52]. Inhalation of large infectious droplets, along with direct contact with secretions or aerosols, facilitates human‐to‐human transmission. Assessing the pandemic potential of H1N1 proves challenging due to limited data; however, epidemiological analyses indicate that its transmission is significantly higher than that of seasonal flu and comparable to past influenza pandemics [53]. The symptoms most commonly documented include fever, cough, and sore throat. Additionally, patients mention experiencing diarrhea, while others report vomiting, while myalgia and joint pains may also be observed.

The majority of deaths are documented in the Americas, while Asia, Europe, and Africa have reported fewer fatalities. The primary cause of most deaths is respiratory failure resulting from severe pneumonia and acute respiratory distress syndrome. In Mexico, a significant portion of deaths occurred in previously healthy individuals aged between 20 and 59 [54]. In other parts of the world, fatalities were predominantly observed in individuals with pre‐existing health issues [55].

The H1N1 influenza pandemic exhibited a spatiotemporal pattern, unfolding in three distinct waves during the spring, summer, and fall of 2009, with notable regional clusters. The initial wave, occurring in April–May, primarily affected the greater Mexico City area and central states. The second wave, which began in June 2009, was limited to the southern states. The third wave began in August, coinciding with students returning from summer break, and persisted through December across Mexico [56].

The respective reproduction numbers were estimated as 1.8–2.1, 1.6–1.9, and 1.2–1.3, during the spring, summer, and fall of 2009. According to an analysis on influenza‐like illness patients in Mexico, the 95% confidence interval for the case fatality ratio (CFR) for the entire pandemic period was estimated to be 1.1%–1.2% [56]. Influenza virus shedding (the time during which a person might be infectious to another person) begins the day before illness onset and can persist for 5–7 days, although some people may shed virus for longer periods [57]. A study conducted in the village of La Gloria reported that 86% of cases were symptomatic and ascertained, with a 95% confidence interval of 69%–100% [53]. It should be noted that the estimation of the asymptomatic ratio according to data derived from small (isolated) regions is not new [58], while the reported ratio necessitates the inclusion of asymptomatic cases in the model. Finally, since the first wave lasted from April to May, the second from June to July, and the third from August to December, we select $u_{F}\left(1,2\right)=1/60$, $u_{F}\left(2,3\right)=1/60$, and $u_{F}\left(f,f^{\prime }\right)=0$, otherwise.

The mapping of these empirical quantities to the model parameters should be interpreted with caution. The reported reproduction‐number estimates correspond to different regional epidemic waves in a spatially heterogeneous setting. In the present finite‐population scenario analysis, we use them as EFS‐specific values of $R_{0}$ for a single homogeneous closed population, which is an additional modeling assumption. Moreover, the reported value of 86% refers to the proportion of cases that were symptomatic and ascertained. This quantity is not automatically identical to the parameter p, which denotes the probability that a new infection is symptomatic. Therefore, in the present illustration, this value is used as a literature motivated proxy for p, and the resulting numerical values should be interpreted as scenario outputs rather than fitted estimates.

The main objective is to assess the disease's severity with respect to the worst, median and best‐case scenarios dictated by the estimated parameter ranges. In this application we use the immunity‐preserving Variant II of Section 2.1.2, since reinfections are not assumed to occur within the pandemic horizon considered. The analysis should be interpreted as a finite‐population H1N1 scenario study for a small closed or semi‐closed subpopulation, where individual interactions and stochastic effects remain relevant. The aim is to illustrate how the proposed framework quantifies expected additional infections and peak infectious burden under parameter values motivated by the H1N1 pandemic. For substantially larger populations, the exact matrix‐based computation becomes more demanding because of the rapid growth of state space. In such cases, Gillespie‐type or Monte Carlo simulations may be used.

According to the above, we set N=50 and consider a time‐varying transmission rate. In addition to the absolute values, emphasis should be placed on the proportion of total and maximum number of infections with respect to the population size. According to the above, we set:

(A) Best case: $R_{0}=(1.8,\;1.6,\;1.2)$, CFR=0.011, while $\gamma =\kappa =\frac{1}{6}$ and p=1,
(B) Median case: $R_{0}=(1.95,\;1.75,\;1.25)$, CFR=0.0115, while $\gamma =\kappa =\frac{1}{6}$ and p=0.86,
(C) Worst case: $R_{0}=(2.1,\;1.9,\;1.3)$, CFR=0.012, while $\gamma =\kappa =\frac{1}{6}$ and p=0.69.

Based on the CFR, where CFR=μ/(γ+μ), we can determine the respective mortality rate μ. So, for the three scenarios we obtain μ=0.0018, μ=0.0019, and μ=0.002. Using the reproduction numbers for the three infection waves and considering Equation (A12) of Appendix B, we can calculate the respective transmission rates. As a result, scenarios A–C are associated with β=(0.303,0.270,0.202), β=(0.329,0.295,0.211), and β=(0.353,0.319,0.219), correspondingly.

Since during the examined period there is no evidence of reinfections of previously infected individuals, we consider that patients receive infection‐induced immunity for the remainder of the pandemic. Thus, we employ the modeling framework presented in Section 2.1.2. It should be underlined that the expected number of symptomatic/asymptomatic infections does not include the initial number of infectious cases.

For both descriptors we examine several initial states aiming to address their influence on the evolution of the pandemic (Tables 2 and 3). It should be noted that in these two tables we examine the same initial states. For instance, state (i,a,r,f)=(1,1,0,1) corresponds to the initial state (i+a,a,r,f)=(2,1,0,1) for the exploration of the maximum number of simultaneously infectious cases.

**TABLE 2:** Expected total number of new symptomatic/asymptomatic infections and standard deviations for five initial states and three epidemic scenarios.

		(1,1,0,1)	(2,1,0,1)	(1,2,0,1)	(2,2,0,1)	(2,2,2,1)
Best case	$E(N^{S})$	21.386	25.156	25.191	27.175	24.660
	$SD(N^{S})$	16.001	14.050	14.037	12.175	11.744
	$E(N^{A})$	0	0	0	0	0
	$SD(N^{A})$	0	0	0	0	0
Median case	$E(N^{S})$	21.042	24.298	24.328	25.890	23.611
	$SD(N^{S})$	14.325	12.175	12.160	10.310	10.070
	$E(N^{A})$	3.425	3.955	3.960	4.215	3.844
	$SD(N^{A})$	2.884	2.693	2.692	2.521	2.432
Worst case	$E(N^{S})$	18.733	21.302	21.325	22.441	20.571
	$SD(N^{S})$	11.807	9.789	9.775	8.177	8.062
	$E(N^{A})$	8.416	9.570	9.581	10.082	9.242
	$SD(N^{A})$	5.730	4.967	4.962	4.370	4.273

**TABLE 3:** Expected maximum number of simultaneously infectious cases, standard deviations, and probabilities that the maximum number exceeds c=5, c=10, and c=20, for five initial states and three epidemic scenarios.

		(2,1,0,1)	(3,1,0,1)	(3,2,0,1)	(4,2,0,1)	(4,2,2,1)
Best case	E(ξ)	8.831	10.463	10.476	11.565	10.815
	SD(ξ)	5.451	4.930	4.930	4.529	4.281
	P(ξ>5)	0.645	0.791	0.792	0.887	0.869
	P(ξ>10)	0.401	0.502	0.503	0.583	0.515
	P(ξ>20)	0.016	0.022	0.022	0.028	0.016
Median case	E(ξ)	9.944	11.610	11.624	12.686	11.839
	SD(ξ)	5.865	5.208	5.207	4.743	4.506
	P(ξ>5)	0.695	0.831	0.832	0.914	0.899
	P(ξ>10)	0.491	0.598	0.599	0.676	0.611
	P(ξ>20)	0.032	0.041	0.041	0.051	0.030
Worst case	E(ξ)	10.997	12.679	12.694	13.721	12.793
	SD(ξ)	6.190	5.407	5.404	4.887	4.667
	P(ξ>5)	0.732	0.861	0.862	0.933	0.921
	P(ξ>10)	0.569	0.676	0.678	0.749	0.689
	P(ξ>20)	0.053	0.067	0.067	0.081	0.050

It is evident that in the worst‐case scenario, a higher proportion of total infections is expected. For instance, for (i,a,r,f)=(1,1,0,1) we expect about 18.733 symptomatic and 8.416 asymptomatic cases (about 27.149 additional infections) which corresponds to more than 50% of the population. On the other hand, for the best‐case scenario and for the same initial state, we observe an expected value of 21.386 symptomatic infections. We also notice that in this case, there are no asymptomatic infections since the symptomatic ratio (p) equals unity. The expected maximum number of simultaneously infectious cases is 8.831 (17.7%) and 10.997 (22.0%) for the best‐ and worst‐case scenario, correspondingly. The median case presents an intermediate between the aforementioned scenarios, where for (i,a,r,f)=(1,1,0,1) we expect about 24 additional infections (48%) with an expected maximum of 9.944 (19.9%) infectious individuals.

Regarding the initial states examined, it is evident that the state (i,a,r,f)=(2,2,0,1) is associated with the highest expected total and peak number of infectious cases across all three scenarios. This can be attributed to the higher number of symptomatic and asymptomatic individuals, combined with the absence of removed individuals, allowing all to contribute to the transmission of the virus. Consequently, the state (i,a,r,f)=(2,2,2,1) results in lower expected infection numbers compared to (i,a,r,f)=(2,2,0,1).

The low number of infectious individuals in the state (i,a,r,f)=(1,1,0,1), may lead to the premature extinction of the disease, which explains why this state is associated with the lowest expected number of infections. Finally, we observe that the state (i,a,r,f)=(1,2,0,1) generates slightly more infections than (i,a,r,f)=(2,1,0,1), even though both are characterized by three infectious individuals. This pattern is consistent across all three scenarios and for both stochastic descriptors. From this, we conclude that the presence of asymptomatic cases is more burdensome for the population under the present settings. This is likely because asymptomatic individuals exhibit only mild or no symptoms and typically do not progress to mortality. As a result, they remain in the infectious state for a longer duration, thereby contributing to sustained transmission of the disease.

## 4 Discussion

In this paper, we introduce a stochastic SuASR framework that accounts for the influence of random external factors. The analysis is based on an extended CTMC that captures the effects of external factors, which can significantly affect the parameters governing the epidemic dynamics. The proposed stochastic framework accounts for two sources of infection, originating from either asymptomatic or symptomatic individuals, leading to more complex and insightful dynamics. Notably, the integration of a Markovian model that incorporates asymptomatic infections along with the impact of external factors constitutes a novel contribution to literature. Recognizing the significant roles both symptomatic and asymptomatic individuals play in disease transmission, we introduce new computational methods for efficiently calculating their joint distribution, which represents the driving force of the model.

By modifying the initially constructed four‐dimensional chain, we derive formulas for computing extreme values, such as the maximum number of simultaneously infectious cases prior to disease eradication. Overall, the maximum number along with the total number of new symptomatic/asymptomatic infections offer a comprehensive depiction of the epidemic's severity, capturing both the total number of infections from each source and the occurrence of extreme cases. The proposed framework comprises two related formulations, allowing either recurrent‐infection dynamics or immunity‐preserving dynamics according to the epidemiological setting.

The computation of the joint distribution for the total number of new symptomatic/asymptomatic infections provides a significant advantage. It enables the derivation of formulas for the marginal distributions of each infection type. Specifically, we have $\tilde{p}(n)=\sum_{m\in {\mathbb{N}}_{0}}\tilde{p}(n,m)$ and $\tilde{p}(m)=\sum_{n\in {\mathbb{N}}_{0}}\tilde{p}(n,m)$, where ${\mathbb{N}}_{0}=\mathbb{N}\cup \left\{0\right\}$. Apparently, the computation of infinite terms is infeasible. However, quantities $\sum_{m=m_{0}+1}^{\infty }\tilde{p}(n,m)$ and $\sum_{n=n_{0}+1}^{\infty }\tilde{p}(n,m)$ become negligible for $m_{0}$ and $n_{0}$ sufficiently large; thus $\sum_{m=m_{0}+1}^{\infty }\tilde{p}(n,m)\approx \varepsilon (m_{0})$ and $\sum_{n=n_{0}+1}^{\infty }\tilde{p}(n,m)\approx \varepsilon \left(n_{0}\right)$. Hence, we obtain

$$\tilde{p}(n)=\underset{m\in {\mathbb{N}}_{0}}{\sum }\tilde{p}(n,m)\approx \overset{m_{0}}{\underset{m=0}{\sum }}\tilde{p}(n,m)+\varepsilon \left(m_{0}\right)\approx \overset{m_{0}}{\underset{m=0}{\sum }}\tilde{p}(n,m),$$

and

$$\tilde{p}(m)=\underset{n\in {\mathbb{N}}_{0}}{\sum }\tilde{p}(n,m)\approx \overset{n_{0}}{\underset{n=0}{\sum }}\tilde{p}(n,m)+\varepsilon \left(n_{0}\right)\approx \overset{n_{0}}{\underset{n=0}{\sum }}\tilde{p}(n,m),$$

that can be used to estimate the respective marginal probability distributions.

In the Methodology section, we present alternative representations for computing the joint distribution, each of which is more suitable depending on the population size (N). Furthermore, we recommend solving the resulting matrix equations, using methods such as LU factorization, without explicitly computing the inverse matrix, in order to minimize the number of operations. Additionally, storing high‐dimensional matrices in sparse format is advisable, as they are predominantly composed of zeros due to their block tridiagonal structure. Adopting this approach significantly reduces the system's memory requirements.

Regarding the Results section, an extensive sensitivity analysis containing several illustrative examples is provided. Emphasis is placed on both the examination of the system's behavior concerning the epidemic's parameters or the transition rates among the EFSs and the application of the methodology based on parameters derived from a real‐world outbreak. Higher transmission (β) and lower recovery (γ,κ) and mortality (μ) rates are associated with prolonged epidemic durations, and consequently greater numbers of total and maximum infections, as infected individuals remain for longer periods in compartments facilitating the spread of the disease. This observation is consistent with the results of [12, 24, 26, 59, 60, 61].

An interesting observation is that it is possible for two epidemic scenarios to differ in such a way that one results in a higher total number of infections while exhibiting a lower peak number of infectious individuals compared to the other. In scenarios where the spread of infection occurs gradually, due to factors such as moderate transmission rates or the implementation of control measures, the epidemic tends to persist over a longer duration. As a result, although the total number of infections may be higher, the infectious burden remains relatively low, producing a flattened epidemic curve. Conversely, in scenarios characterized by rapid transmission and limited intervention, the number of infectious individuals can rise sharply to a higher peak, but the epidemic may subside quickly due to rapid recovery or the depletion of susceptible individuals, ultimately leading to a lower total number of cases. These distinctions underscore the importance of considering both the peak burden and the total disease impact when evaluating and comparing epidemic outcomes.

The necessity of investigating epidemic dynamics through stochastic, and not deterministic models is highlighted. In cases of bimodal distributions, the information provided by the deterministic trajectories given by an ODE system is insufficient. The existence of two peaks signifies the existence of scenarios where the epidemic may die out prematurely or persist for an extended period. These scenarios, accompanied by high standard deviations, necessitate more attention and close inspection of the epidemic's evolution due to significant uncertainty. Public health authorities can use this information to adjust the implementation of control measures and help contain the spread of the disease.

Finally, it is worth noting that certain modeling efforts have explored transition periods with generally distributed durations using piecewise‐deterministic Markov processes [62, 63]. However, beyond these approaches there is a notable lack of analysis on the impact of non‐exponential transition times within the Markov framework. To address this gap, we propose integrating semi‐Markov processes, which allow for more flexible sojourn time distributions and may offer valuable insights to existing literature. Finally, emphasis could be placed on change point analysis (CPA) applied to real epidemic data from novel disease outbreaks. CPA enables the detection of shifts in the dynamics of the outbreak [64, 65, 66], allowing us to identify transitions among EFSs. This, in turn, will facilitate the development of a statistical estimation approach suitable for both offline and online prediction.

## Funding

This research was funded by the A. G. Leventis Foundation through a doctoral studies grant.

## Ethics Statement

This study did not involve human participants, human material, or identifiable personal data. It is based on mathematical modeling and publicly available/aggregate data.

## Conflicts of Interest

The authors declare no conflicts of interest.

## Supporting information


**Data S1**: sim70796‐sup‐0001‐Supinfo.zip.

## Data Availability

The code used for the simulation study is publicly available at https://github.com/VasilisPapageorgiou/markovian‐suasr.

## Appendix A

Aiming to prove formula (19) and in order to construct an algorithm for the computation of the $\tilde{p}\left(n,m\right)$ vector, we define the generating functions $\varphi_{i,a,r,f}\left(z,s\right)$ for |z|,|s|≤1 and $\left(i,a,r,f\right)\in A_{T}$, where based on first‐step analysis [67], we obtain the formula

(A1)
φi,a,r,f(z,s)=zp(f)u˜i,a,r,fβ(f)Ni,a,rφi+1,a,r,f(z,s)(1−δN−i−a−r,0)+s1−p(f)u˜i,a,r,fβ(f)Ni,a,rφi,a+1,r,f(z,s)(1−δN−i−a−r,0)+μi(f)u˜i,a,r,fφi−1,a,r+1,f(z,s)(1−δi0)+γi(f)u˜i,a,r,fφi−1,a,r,f(z,s)(1−δi0)+κa(f)u˜i,a,r,fφi,a−1,r,f(z,s)(1−δa0)+∑f′=1f′≠fFuF(f,f′)u˜i,a,r,fφi,a,r,f′(z,s).

After multiplying both sides by the denominator ${\tilde{u}}_{i,a,r,f}$ and expressing (A1) in matrix form, we obtain

(A2)
$$\tilde{U}\left(z,s\right)\varphi \left(z,s\right)=-\left(\delta_{i,1}\delta_{a,0}\left(\mu_{1}\left(f\right)+\gamma_{1}\left(f\right)\right)+\delta_{i,0}\delta_{a,1}\;\kappa_{1}\left(f\right)\right)1_{\left|A_{T}\right|},$$

considering that for i=1 and a=0,

(A3)
zp(f)β(f)N1,0,rφ2,0,r,f(z,s)(1−δN−1−r,0)+∑f′=1f′≠fFuF(f,f′)φ1,0,r,f′(z,s)+s(1−p(f))β(f)N1,0,rφ1,1,r,f(z,s)(1−δN−1−r,0)−u˜1,0,r,fφ1,0,r,f(z,s)=−(μ1(f)+γ1(f)),1≤f≤F,

as $\varphi_{0,0,r,f}\left(z,s\right)=1$, due to disease's extinction. The same applies for the case i=0 and a=1,where

(A4)
zp(f)β(f)N0,1,rφ1,1,r,f(z,s)(1−δN−1−r,0)+∑f′=1f′≠fFuF(f,f′)φ0,1,r,f′(z,s)+s(1−p(f))β(f)N0,1,rφ0,2,r,f(z,s)(1−δN−1−r,0)−u˜0,1,r,fφ0,1,r,f(z,s)  =−κ1(f),1≤f≤F.

We note that the polynomial matrix $\tilde{U}\left(z,s\right)=\left\{{\tilde{u}}_{\left(i,a,r,f\right),\left(i^{\prime },a^{\prime },r^{\prime },f^{\prime }\right)}\left(z,s\right):\left(i,a,r,f\right),\left(i^{\prime },a^{\prime },r^{\prime },f^{\prime }\right)\in A_{T}\right\}$, has the form

(A5)
$$\begin{array}{cc} & {\tilde{u}}_{\left(i,a,r,f\right),\left(i^{\prime },a^{\prime },r^{\prime },f^{\prime }\right)}\left(z,s\right)\\ & \;=\left\{\begin{array}{ll} -{\tilde{u}}_{i,a,r,f},\; & \left(i^{\prime },a^{\prime },r^{\prime },f^{\prime }\right)=\left(i,a,r,f\right),\\ zp\left(f\right){\left(\frac{\beta \left(f\right)}{N}\right)}_{i,a,r},\; & \left(i^{\prime },a^{\prime },r^{\prime },f^{\prime }\right)=\left(i+1,a,r,f\right),\\ s\left(1-p\left(f\right)\right){\left(\frac{\beta \left(f\right)}{N}\right)}_{i,a,r},\; & \left(i^{\prime },a^{\prime },r^{\prime },f^{\prime }\right)=\left(i,a+1,r,f\right),\\ \gamma_{i}\left(f\right),\; & \left(i^{\prime },a^{\prime },r^{\prime },f^{\prime }\right)=\left(i-1,a,r,f\right),\\ \kappa_{a}\left(f\right),\; & \left(i^{\prime },a^{\prime },r^{\prime },f^{\prime }\right)=\left(i,a-1,r,f\right),\\ \mu_{i}\left(f\right),\; & \left(i^{\prime },a^{\prime },r^{\prime },f^{\prime }\right)=\left(i-1,a,r+1,f\right),\\ u_{F}\left(f,f^{\prime }\right),\; & \left(i^{\prime },a^{\prime },r^{\prime },f^{\prime }\right)=\left(i,a,r,f^{\prime }\right),f\neq f^{\prime },\\ 0,\; & \text{otherwise}. \end{array}\right. \end{array}$$

ALGORITHM 1 Joint Probability Distribution of the Total Number of New Symptomatic/Asymptomatic Cases.

The column vector φ(z,s) includes all the generating functions corresponding to the set of the system's transient states. By setting z=s=0 in (A2), we generate the vector of the joint distribution $\tilde{p}(0,0).$ Algorithm 1 presents the calculation of the three‐dimensional tensor $\tilde{p}(n,m)$, based on the recursive formula (19) presented in the Remark. The rows and columns of the tensor correspond to the total number of new symptomatic and asymptomatic cases, respectively, while the third dimension is associated with the selected current state of the system.

Algorithm 1 is presented for Variant I. For Variant II, the same computational procedure applies, with the only modifications concerning the recovery‐related transition blocks, which must be defined according to the immunity‐preservingformulation of Section 2.1.2.

## Appendix B

In the experimental part of the present paper, we examine the spread of the H1N1 influenza pandemic of 2009, using the model variant presented in Section 2.1.2. In this section, the selection of realistic values for the system's parameters is necessary. The transmission rate (β) can be retrieved from the widely utilized basic reproduction number ${(R}_{0})$. This index represents the expected number of secondary infections generated by an already infected individual inside a nearly susceptible population.

We can derive a formula for this index based on the methodology of the next‐generation matrix which was developed by [68] and was popularized by [69]. Following the process described in [39, 69], we first define the disease‐free equilibrium (DFE) $Y^{0}=\left(S^{0},\;I^{0},A^{0}\right)=(N,0,0).$ This vector is associated with the absence of the virus from the examined population. We notice that the presented epidemiological scheme contains a system of four differential equations. However, we present only three components for $Y^{0}$. This modification derives from the fact that the number of removed cases is a linear combination of the other three states, since the examined population is closed.

Next, we select the vector X=(I,A) which contains the two states of the system which are associated with the existence and spread of the virus. Let ℱ(X) and 𝒱(X) be 2×1 vectors whose k‐th entry exhibits the rate of new infections entering state k and the transfer rate out of state k by non‐infection means, respectively. As a result,

(A6)
$${\dot{X}}^{T}=ℱ\left(X\right)-𝒱\left(X\right),$$

where

(A7)
$$ℱ\left(X\right)={\left(p\frac{\beta }{N}S(t)(I(t)+A(t)),\;(1-p)\frac{\beta }{N}S(t)(I(t)+A(t))\right)}^{T},$$

and

(A8)
$$𝒱\left(X\right)={\left(\left(\gamma +\mu )I(t),\;\kappa A(t\right)\right)}^{T}.$$

Now, the next‐generation matrix is given by the product $FV^{-1}$. The matrices F and V are the Jacobians of ℱ(X) and 𝒱(X) evaluated at the disease‐free equilibrium $Y^{0}$. Both F and V are 2×2 matrices, given by

(A9)
$$F=\left(\begin{array}{cc} \begin{array}{c} p\beta \\ (1-p)\beta \end{array} & \begin{array}{c} p\beta \\ (1-p)\beta \end{array} \end{array}\right),$$

and

(A10)
$$\;V=\;\left(\begin{array}{cc} \begin{array}{c} \gamma +\mu \\ 0 \end{array} & \begin{array}{c} 0\\ \kappa \end{array} \end{array}\right).$$

Apparently, matrix V is invertible, where the inverse matrix has the form

(A11)
$$V^{-1}=\;\left(\begin{array}{cc} \begin{array}{c} \frac{1}{\gamma +\mu }\\ 0 \end{array} & \begin{array}{c} 0\\ \frac{1}{\kappa } \end{array} \end{array}\right).$$

Finally, $R_{0}$ is the spectral radius of the next‐generation matrix $FV^{-1}$, leading to the expression

(A12)
$$R_{0}=\rho \left(FV^{-1}\right)=(1-p)\frac{\beta }{\kappa }+p\frac{\beta }{\gamma +\mu },$$

where ρ(A) stands for the spectral radius of matrix A. Considering that parameters γ, κ, p, μ, and $R_{0}$ are known, we can determine the appropriate β value.
